# A *Pseudomonas aeruginosa* small RNA regulates chronic and acute infection

**DOI:** 10.1038/s41586-023-06111-7

**Published:** 2023-05-24

**Authors:** Pengbo Cao, Derek Fleming, Dina A. Moustafa, Stephen K. Dolan, Kayla H. Szymanik, Whitni K. Redman, Anayancy Ramos, Frances L. Diggle, Christopher S. Sullivan, Joanna B. Goldberg, Kendra P. Rumbaugh, Marvin Whiteley

**Affiliations:** 1 School of Biological Sciences, Georgia Institute of Technology, Atlanta, GA USA; 2 Emory-Children’s Cystic Fibrosis Center, Atlanta, GA USA; 3 Center for Microbial Dynamics and Infection, Georgia Institute of Technology, Atlanta, GA USA; 4 Department of Surgery, Texas Tech University Health Sciences Center, Lubbock, TX USA; 5 Department of Immunology and Molecular Microbiology, Texas Tech University Health Sciences Center, Lubbock, TX USA; 6 Burn Center of Research Excellence, Texas Tech University Health Sciences Center, Lubbock, TX USA; 7 Department of Pediatrics, Division of Pulmonary, Asthma, Cystic Fibrosis, and Sleep, Emory University School of Medicine, Atlanta, GA USA; 8 Department of Molecular Biosciences, The University of Texas at Austin, Austin, TX USA; 9 Present Address: Division of Clinical Microbiology, Department of Laboratory Medicine and Pathology, Mayo Clinic, Rochester, MN USA; 10 Present Address: Department of Biological Sciences, Binghamton University, Binghamton, NY USA

**Keywords:** Bacterial pathogenesis, Small RNAs, Bacterial genetics, Pathogens

## Abstract

The ability to switch between different lifestyles allows bacterial pathogens to thrive in diverse ecological niches^1,2^. However, a molecular understanding of their lifestyle changes within the human host is lacking. Here, by directly examining bacterial gene expression in human-derived samples, we discover a gene that orchestrates the transition between chronic and acute infection in the opportunistic pathogen *Pseudomonas aeruginosa*. The expression level of this gene, here named *sicX*, is the highest of the *P.* *aeruginosa* genes expressed in human chronic wound and cystic fibrosis infections, but it is expressed at extremely low levels during standard laboratory growth. We show that *sicX* encodes a small RNA that is strongly induced by low-oxygen conditions and post-transcriptionally regulates anaerobic ubiquinone biosynthesis. Deletion of *sicX* causes *P.* *aeruginosa* to switch from a chronic to an acute lifestyle in multiple mammalian models of infection. Notably, *sicX* is also a biomarker for this chronic-to-acute transition, as it is the most downregulated gene when a chronic infection is dispersed to cause acute septicaemia. This work solves a decades-old question regarding the molecular basis underlying the chronic-to-acute switch in *P.* *aeruginosa* and suggests oxygen as a primary environmental driver of acute lethality.

## Abstract (web summary)

A study examining bacterial gene expression in human-derived samples identifies a gene encoding a small RNA and describes how it orchestrates the transition between chronic and acute infection in *Pseudomonas aeruginosa*.

## Main

Many pathogenic bacteria can colonize their hosts and persist chronically. In some cases, bacteria disseminate from the initial infection sites to other body parts, resulting in acute systemic diseases. A central question in biology is to understand the molecular mechanisms and environmental cues controlling this lifestyle switch. The opportunistic human pathogen *P.* *aeruginosa* can cause both acute and chronic infections that are notoriously difficult to treat. *P.* *aeruginosa* exists as single cells (planktonic lifestyle) or matrix-enclosed aggregates (biofilm lifestyle), which are considered to favour acute and chronic infections, respectively. Therefore, laboratory studies have traditionally focused on probing the biofilm–planktonic transition of *P.* *aeruginosa* in vitro, with the goal to understand this pathogen’s lifestyle changes in humans. Decades of work have shown how global regulatory systems such as the second messenger cyclic di-GMP^3–5^ and the Gac–Rsm system^2,6–12^ control the biofilm–planktonic transition of *P.* *aeruginosa* in vitro, providing critical insights into the life history of this bacterium. However, so far, it has remained elusive how *P.* *aeruginosa* responds to environmental cues and accordingly switches infection lifestyles within the mammalian host. In this study, we addressed this gap in knowledge by leveraging *P.* *aeruginosa* transcriptomes acquired from human-derived samples to discover and mechanistically characterize a new small RNA, termed sRNA inducer of chronic infection X (SicX), that governs the *P.* *aeruginosa* chronic or acute decision during mammalian infection.

## High expression of *PA1414* in humans

**Fig. 1:**
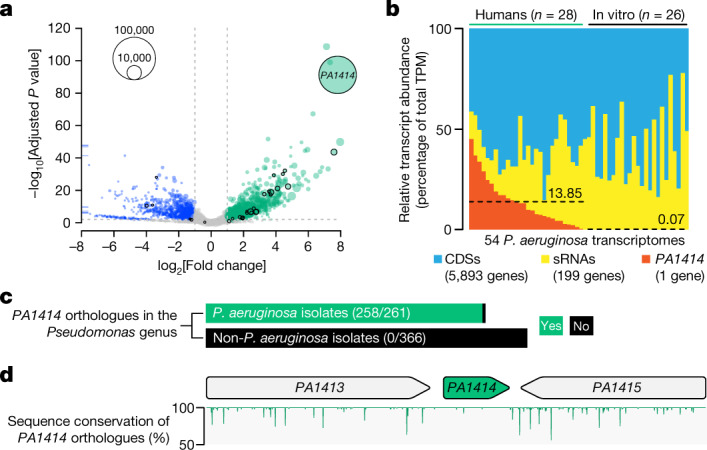
The expression level of *PA1414* is the highest of the *P.* *aeruginosa* genes expressed during human chronic infections. **a**, Differential gene expression of *P.* *aeruginosa* between human infections and common in vitro conditions. For each gene, the log_2_[fold change] is plotted against the Wald test *P* value. Bubbles with black borders highlight the 30 signature genes of *P.* *aeruginosa* human infection. Bubble size indicates mRNA read abundance during human infections. Blue bars on the *y* axis indicate genes (with negative log_2_[fold change] values) outside the scale range. Dashed grey lines indicate the cutoffs (−log_10_[adjusted *P* value] > 2, |log_2_[fold change]| > 1) for identifying differentially expressed genes. The upregulated (green) and downregulated (blue) genes in humans are colour-coded differently. Grey bubbles indicate genes that are not differentially expressed. **b**, Relative transcript abundance (TPM) of *PA1414* compared to those of all other protein-coding sequences (CDSs) and non-coding sRNAs in 54 transcriptomes examined in **a**, sorted from the highest to the lowest *PA1414* TPM. Average *PA1414* TPM is indicated with dashed lines. **c**, *PA1414* orthologues are found only in *P.* *aeruginosa*. Green and black bars indicate the presence and absence of orthologues, respectively. **d**, DNA sequence conservation of *PA1414* and its neighbouring genes among 258 *PA1414*-containing *P.* *aeruginosa* isolates. Below, the percentage of orthologues identical to the *PA1414* allele from PA14 at each nucleotide is shown. Source Data

**Extended Data Fig. 1:**
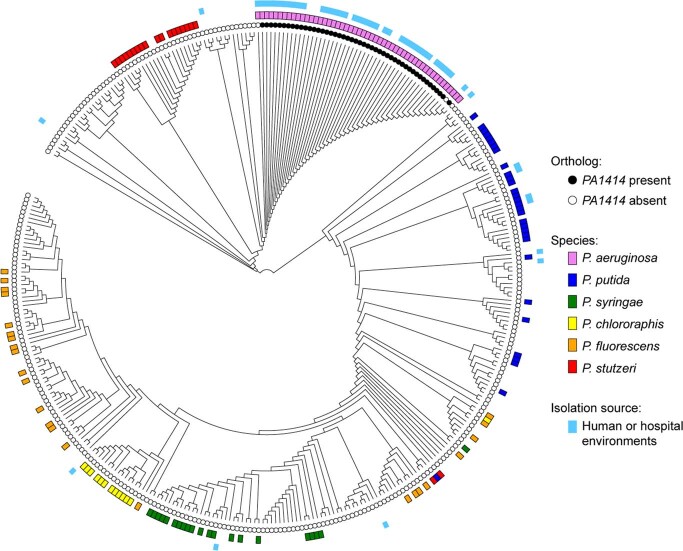
*PA1414* orthologs are restricted in *P. aeruginosa*. The maximum-likelihood tree is built with 16s rDNA sequences from representative strains across the *Pseudomonas* genus. Clusters of abundant species are indicated with colored boxes. Strains that were reported to have been isolated from human or hospital environments are highlighted.

We previously obtained high-resolution *P.* *aeruginosa* transcriptomes from human infection samples, including sputum samples from patients with cystic fibrosis and debridement samples from patients with chronic wounds^13,14^. Using machine learning methods, we identified 30 *P.* *aeruginosa* genes whose expression levels collectively differentiate *P.* *aeruginosa* growth in humans from that in the laboratory^13^. More than half of these genes are uncharacterized, highlighting a substantial gap in knowledge of *P.* *aeruginosa* human infection biology. Included in these genes of unknown function is *PA1414* (locus tag in *P.* *aeruginosa* strain PAO1), whose expression level in humans was 222-fold higher than that in the laboratory, and its expression level is the highest of the *P.* *aeruginosa* genes expressed during human infection (Fig. 1a). We next compared the relative abundance (transcripts per million (TPM)) of *PA1414* transcripts with those of other protein-coding (5,893 genes) and non-coding (199 sRNAs)^15^ transcripts (Fig. 1b). On average, *PA1414* transcripts represented 13.85% of the TPM in *P.* *aeruginosa* transcriptomes acquired from human chronic infection samples, and notably, they constituted almost 50% of the total TPM in some cases. However, the level of *PA1414* expression was extremely low in *P.* *aeruginosa* grown under standard in vitro conditions. *PA1414* is a small gene (234 base pairs long) of unknown function. Examination of 261 complete *P.* *aeruginosa* genomes identified 258 *PA1414* orthologues (Fig. 1c and Extended Data Fig. 1), and no homologues were identified in other *Pseudomonas* species or other organisms. The DNA sequences of *PA1414* orthologues including their upstream promoter regions are highly conserved (Fig. 1d), suggesting that *PA1414* has a conserved function and the regulation of its expression is universal across *P.* *aeruginosa* isolates.

## Low oxygen induces *PA1414* expression

**Fig. 2:**
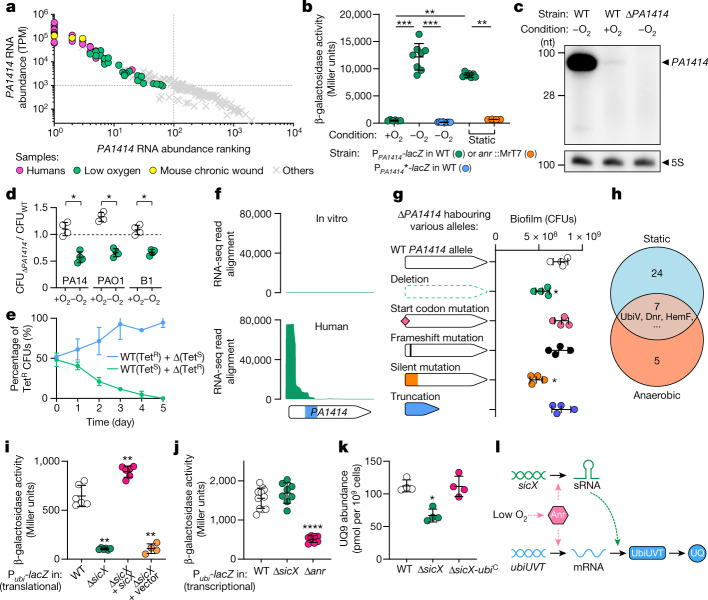
*PA1414* encodes an oxygen-responsive sRNA that governs anaerobic ubiquinone biosynthesis. **a**, *PA1414* RNA abundance (TPM) and the corresponding ranking (out of all protein-coding genes and sRNAs) in 202 *P.* *aeruginosa* transcriptomes. Dashed lines indicate the cutoffs (*PA1414* TPM > 10^3^, *PA1414* ranking < 10^2^) for identifying transcriptomes with high *PA1414* expression. **b**, β-galactosidase assay examining the induction of *PA1414* under anaerobic and static conditions. P_*PA1414*_-*lacZ*, *lacZ* transcriptionally fused to *PA1414* promoter; P_PA1414_*, *PA1414* promoter containing mutations in the Anr-binding motif; MrT7, MAR2xT7 transposon. *n* ≥ 4 independent experiments. **c**, Northern blot analysis of *PA1414* expression. Estimated size (nucleotides, nt) are indicated on the left. 5S rRNA served as a loading control. For gel source data, see Supplementary Fig. 1. **d**, Colony biofilm growth of Δ*PA1414* and WT in different *P.* *aeruginosa* strain backgrounds. The presence and absence of oxygen are indicated. The CFU of Δ*PA1414* was normalized against the CFU of the WT in each experiment (*n* = 4). A dashed line highlights the point where the CFU ratio = 1. **e**, Percentage of tetracycline-resistant (Tet^R^) cells before and after daily passages under anaerobic conditions (*n* = 3). Tet^S^, tetracycline sensitive; Δ, Δ*PA1414*. **f**, RNA-seq reads aligned to the *PA1414* locus during standard in vitro growth and human infections. Blue shade indicates Rho-independent terminator in *PA1414*. **g**, Colony biofilm growth of Δ*sicX* harbouring different *sicX* mutations under anaerobic conditions. *n* = 4 independent experiments. **h**, Comparative proteomic study identifies the targets of SicX under both static and anaerobic conditions. **i**, β-galactosidase assay evaluating the translational control of SicX on *ubiUVT* under anaerobic conditions (*n* ≥ 4). **j**, β-galactosidase assay evaluating the transcription of *ubiUVT* in the absence of SicX or Anr under static conditions (*n* ≥ 8). **k**, Quantification of anaerobic UQ9 synthesis in different strains (*n* = 4). **l**, A model of dual regulation of *ubiUVT* expression by Anr and SicX. Error bars represent standard deviation from the mean. Significant differences (compared to the WT) are indicated with asterisks (*P < 0.05; **P < 0.01; ***P < 0.001; ****P < 0.0001; two-tailed Mann–Whitney test). Source Data

**Extended Data Fig. 2:**
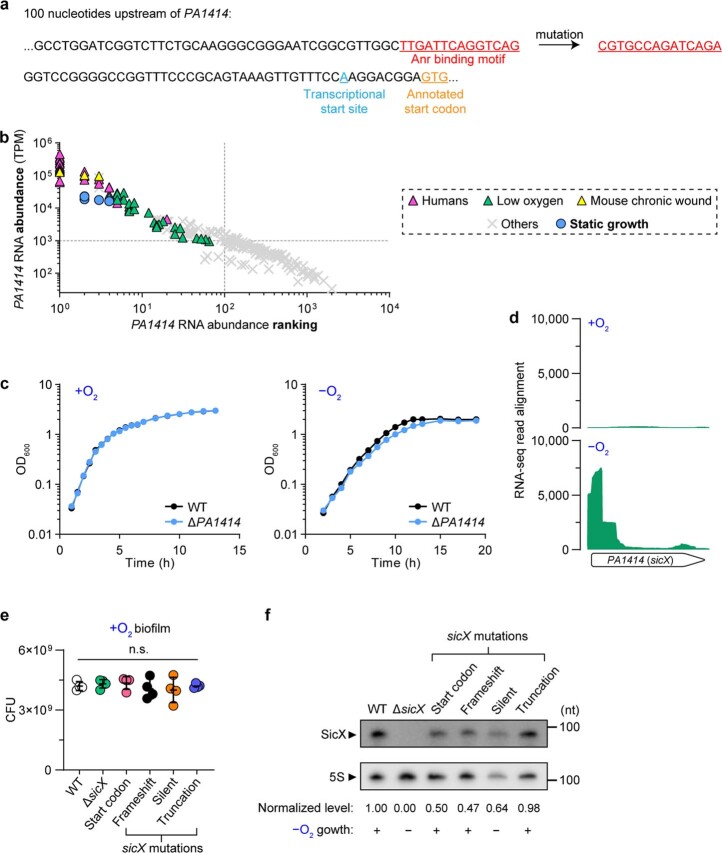
The expression of *PA1414* under low oxygen conditions. **a**. Schematic of mutagenesis of the Anr binding motif. **b**. Figure 2a with the addition of five in vitro static growth transcriptomes (blue circles; the RNA-seq libraries were prepared following the same method used in our study of *P. aeruginosa* transcriptomes in humans). **c**. Planktonic growth (OD_600_) of WT and Δ*PA1414* in the presence or absence of oxygen. Mean values from 3 independent experiments are shown. **d**. RNA-seq reads aligned to the *PA1414* locus (*sicX*) during in vitro aerobic and anaerobic growth. **e**. Colony biofilm growth of Δ*sicX* harboring different *sicX* mutations (as illustrated in Fig. 2g) under aerobic conditions. Error bars represent standard deviation from the mean. *n* = 4 independent experiments. Two-tailed Mann-Whitney test comparing WT and each other strain was performed (n.s., not significant). **f**. Northern blot analyses on different *sicX* alleles as depicted in Fig. 2g. The sRNA abundance was estimated by first normalizing against the 5S rRNA (loading control) and next comparing to the WT level. Anaerobic growth fitness was indicated with + (no defect) and − (defect). For gel source data, see Supplementary Fig. 1.

*P.* *aeruginosa* can thrive in diverse ecological niches within the human body. To understand what environmental cues drive *PA1414* expression, we examined 202 publicly available *P.* *aeruginosa* transcriptomes acquired across a range of environments (Fig. 2a and Supplementary Table 1). We found that *PA1414* was expressed at high levels under low-oxygen and anaerobic conditions. Consistent with this finding, an early microarray study also showed that *PA1414* is induced during anaerobic growth^16^. Moreover, a consensus binding motif for the global anaerobic transcriptional regulator, Anr^16^, was identified upstream of *PA1414* (Extended Data Fig. 2a). Using a *lacZ* reporter that quantifies transcription of *PA1414*, we showed that indeed this gene was induced by 25-fold under anaerobic conditions, and mutagenesis of Anr or the binding motif abolished *PA1414* expression (Fig. 2b). *PA1414* was also expressed at an approximately 18-fold higher level in static cultivation (Fig. 2b), indicating that strict anaerobic conditions are not required for its induction. In addition, northern blot analysis provided direct evidence that *PA1414* transcripts were highly abundant during anaerobic but not aerobic growth (Fig. 2c). To directly compare *PA1414* expression levels in humans and in low-oxygen environments, we conducted RNA-sequencing (RNA-seq) experiments on *P.* *aeruginosa* during in vitro static growth (a low-oxygen condition), following the same library preparation method used in our study of *P.* *aeruginosa* transcriptomes in humans^14^. We found that although under low-oxygen conditions *PA1414* transcript abundance (TPM) was sevenfold lower than that during human infections, *PA1414* is among the three genes expressed at the highest levels (out of approximately 6,000 protein-coding and sRNA genes), supporting the notion that a low oxygen level is indeed a primary driver for its high expression level (Extended Data Fig. 2b). Given that *P.* *aeruginosa* often encounters low-oxygen environments during human chronic infection^17,18^, these data indicate that *P.* *aeruginosa* responds to this host environmental cue by increasing the expression level of *PA1414*.

We next considered whether *PA1414* affects the anaerobic physiology of *P.* *aeruginosa*. For these experiments, we deleted *PA1414* from the *P.* *aeruginosa* strain PA14 (the corresponding locus tag in this strain is *PA14_46160*) and compared the growth of Δ*PA1414* to that of wild-type (WT) *P.* *aeruginosa* under aerobic and anaerobic conditions (Fig. 2d). Using a colony biofilm assay, we found that whereas deletion of *PA1414* did not negatively affect the aerobic growth of *P.* *aeruginosa*, Δ*PA1414* exhibited growth yields approximately half that of WT under anaerobic conditions with nitrate as the alternative electron acceptor (Fig. 2d). Similar observations were made in different *P.* *aeruginosa* strain backgrounds including a cystic fibrosis clinical isolate, B1 (Fig. 2d; ref. ^19^). Although we showed that *PA1414* also played a minor role in anaerobic planktonic growth (Extended Data Fig. 2c), competition experiments in which WT and Δ*PA1414* were co-cultured planktonically (at a 1:1 starting ratio) and passaged daily under anaerobic conditions revealed that WT outcompeted Δ*PA1414* after day 3 (Fig. 2e).

## *PA1414* encodes an sRNA, SicX

We next investigated the molecular mechanism by which *PA1414* controls anaerobic growth. Although annotated as a protein-coding gene, we discovered that during human infection *PA1414* transcripts were restricted to the 5′ end (Fig. 2f), and a similar pattern was also observed under anaerobic conditions (Extended Data Fig. 2d). Clear enrichment of short transcripts encompassing the annotated start codon raised the question of whether *PA1414* encodes a functional protein or a small regulatory RNA (sRNA). To address this question, we introduced different mutations in *PA1414* and assessed whether they could restore the growth yield defect of the Δ*PA1414* colony biofilm under anaerobic conditions (Fig. 2g and Extended Data Fig. 2e). We found that a start codon or frame-shift mutation (to prevent protein synthesis) did not affect the function of *PA1414*, whereas synonymous substitutions in the highly transcribed region (to disrupt potential interactions of sRNA with target mRNAs or RNA-binding proteins) abolished its function. Moreover, a truncated allele encompassing only the highly transcribed 5′ region was sufficient to restore the anaerobic growth defect in Δ*PA1414*, further highlighting this functional region of *PA1414*. Through northern blot analyses, we showed that the observed growth differences were not due to differential sRNA abundance (Extended Data Fig. 2f). Thus, we conclude that *PA1414* functions as an sRNA (hereafter referred to as SicX) that is not essential for but promotes *P.* *aeruginosa* anaerobic growth in vitro.

## SicX regulates anaerobic respiration

As sRNAs often exert their regulation post-transcriptionally^20–22^, we carried out quantitative shotgun proteomics to identify targets of SicX. Here, proteomes of WT *P.* *aeruginosa* and Δ*sicX* were obtained under two growth conditions in which *sicX* is expressed at a high level: low-oxygen (that is, static growth) and strict anaerobic conditions. We identified seven proteins (including three with known functions) exhibiting different abundances between WT and Δ*sicX* under both growth conditions (Fig. 2h and Supplementary Table 2). One of these proteins is UbiV, which is encoded by the *ubiUVT* operon^23^. This operon encodes proteins responsible for the anaerobic biosynthesis of ubiquinone, an essential electron carrier for respiratory metabolism in proteobacteria^23^. Although the other two proteins encoded by this operon, UbiU and UbiT, were not in the seven proteins identified with our stringent criteria, further investigation revealed that both of these proteins also showed significantly reduced levels in Δ*sicX* compared to WT *P.* *aeruginosa* during static growth (Supplementary Table 2). Using a *lacZ* translational reporter of the *ubiUVT* operon, we showed that *sicX* deletion indeed reduced the translational activity of *ubiUVT* by sixfold under anaerobic conditions, and genetic complementation with *sicX* in *trans* restored *ubiUVT* translation (Fig. 2i). Moreover, SicX did not affect the transcription of *ubiUVT* (Fig. 2j). To provide direct evidence of how SicX regulates anaerobic respiration, we quantified the ubiquinone (more specifically, UQ9) abundance in WT and Δ*sicX* under anaerobic conditions by liquid chromatography–mass spectrometry, and indeed, Δ*sicX* exhibited a twofold reduction in UQ9 compared to WT (Fig. 2k). Therefore, we concluded that SicX post-transcriptionally induces the expression of UbiUVT. Our finding also provides insights into the increased abundance of Dnr and HemF in Δ*sicX* (Fig. 2h), as Dnr can induce the denitrification pathway^16^ to alleviate the anaerobic stress in Δ*sicX*, and the expression of HemF is Dnr dependent^24^. Notably, we found that the transcription of *ubiUVT* is under the control of Anr (Fig. 2j). Together, these results support a model in which Anr activates the transcription of *sicX* and *ubiUVT* in response to oxygen deprivation. In turn, SicX post-transcriptionally induces ubiquinone biosynthesis to shape the anaerobic physiology of *P.* *aeruginosa* (Fig. 2l).

**Extended Data Fig. 3:**
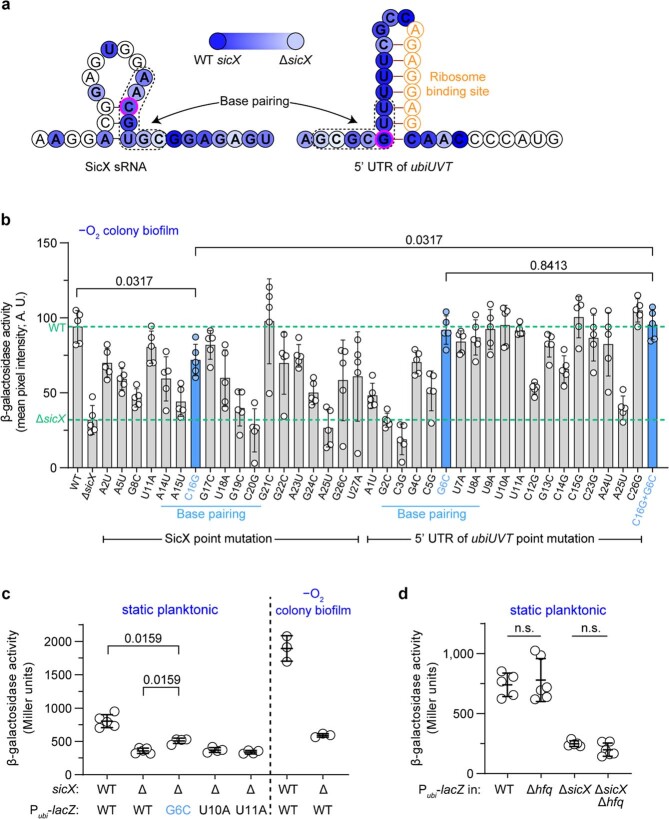
A proposed base-pairing interaction between SicX and the 5’ UTR of *ubiUVT*. **a**. Predicted base-pairing between SicX and *ubiUVT* mRNA. Nucleotides highlighted in bold were subject to point mutations (A → U, U → A, G → C, C → G), which were assessed via the *ubiUVT*-*lacZ* translational reporter. Colony biofilms grown under anaerobic conditions were assessed for β-galactosidase activities as described^46^. Heatmap displays the mean values. Nucleotides subject to compensatory base changes are highlighted with magenta borders. Predicted base-pairing regions are highlighted with dashed borders. **b**. β-galactosidase activities (individual values) of the experiment shown in (A) quantified as described^46^. Potential compensatory mutations are highlighted in blue. Green dashed lines indicate the mean β-galactosidase activities in WT and Δ*sicX*. A. U., arbitrary unit. *n* = 5 independent experiments. **c**. β-galactosidase assays evaluating whether 5’ UTR mutations can alter *ubiUVT* translation independently of SicX during static growth. WT and Δ*sicX* harboring the *ubiUVT-lacZ* reporter (grown as anaerobic colony biofilms) were also assessed using this assay. *n* ≥ 3 independent experiments. **d**. β-galactosidase assays indicating that the presence or absence of Hfq does not affect the induction of *ubiUVT* translation by SicX. *n* ≥ 5 independent experiments. Error bars represent standard deviation from the mean. Two-tailed Mann-Whitney test was performed for **b**–**c** (n.s., not significant).

**Extended Data Fig. 4:**
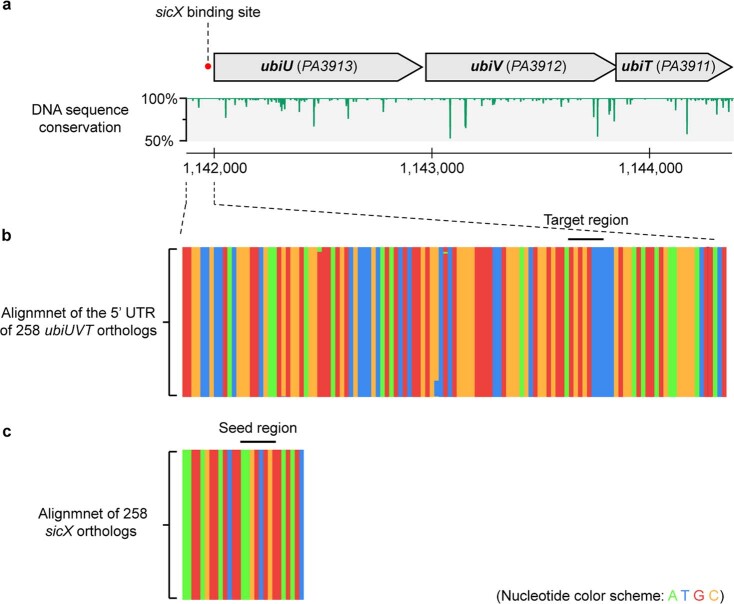
DNA sequence conservation of the *ubiUVT* operon. **a**. The percent identity of 258 *ubiUVT* orthologs (compared to the *ubiUVT* allele from PA14) at the single-nucleotide scale. The *sicX* binding site at the 5’ UTR is highlighted. **b**. DNA sequence alignment of the upstream intergenic region of 258 *ubiUVT* orthologs. **c**. DNA sequence alignment of 258 *sicX* orthologs (only the sRNA region is shown). The seed region in SicX and the target region in the 5’ UTR of *ubiUVT* are indicated.

We next explored how SicX activates the translation of *ubiUVT* mRNA. In silico analysis through IntaRNA^25^ identified a putative base-pairing interaction between SicX and the 5′ untranslated region (UTR) of the *ubiUVT* operon (Extended Data Fig. 3a). Secondary structures predicted with Mfold^26^ indicate that a stem–loop within the 5′ UTR of *ubiUVT* occludes the ribosome-binding site of *ubiU*, whereas base pairing with SicX has the potential to liberate the ribosome-binding site for translation initiation. To experimentally test this, we carried out extensive mutational analyses of *sicX* and the 5′ UTR of *ubiUVT*, and the functional outcome of each point mutation (37 in total) was assessed through a *lacZ* reporter translationally fused to *ubiUVT* (Extended Data Fig. 3a,b). In concordance with in silico prediction, we identified mutations within the SicX seed region (for base pairing with the *ubiUVT* mRNA) and the target region in the 5′ UTR of *ubiUVT* that led to reduced translational activation of *ubiUVT*, whereas mutations in other regions generally had limited effects (Extended Data Fig. 3a,b). Moreover, a point mutation in the seed region of SicX that reduced *ubiUVT* translation can be partially restored by re-establishing base pairing in the 5′ UTR of *ubiUVT* (Extended Data Fig. 3b). However, it should be noted that the observed translational restoration may partly result from the mutation in the 5′ UTR of *ubiUVT*, which increased *ubiUVT* translation even in the absence of SicX (Extended Data Fig. 3c), probably by destabilizing the stem–loop. Finally, we showed that the RNA chaperone Hfq^27^ is not required for the induction of *ubiUVT* translation by SicX (Extended Data Fig. 3d). Although Hfq is known to facilitate the interactions between sRNAs and target mRNAs in many cases, Hfq-independent sRNA–mRNA interactions have been well documented^28–30^. On the basis of our genetic results, we propose an Hfq-independent base-pairing model for SicX stimulation of *ubiUVT* translation, which will be tested in the future. Notably, the predicted base-pairing regions in SicX and the 5′ UTR of *ubiUVT* are identical across all examined *P.* *aeruginosa* genomes (Extended Data Fig. 4a–c), and thus this mechanism of regulation may be universal in *P.* *aeruginosa*.

## SicX governs the chronic-to-acute switch

**Fig. 3:**
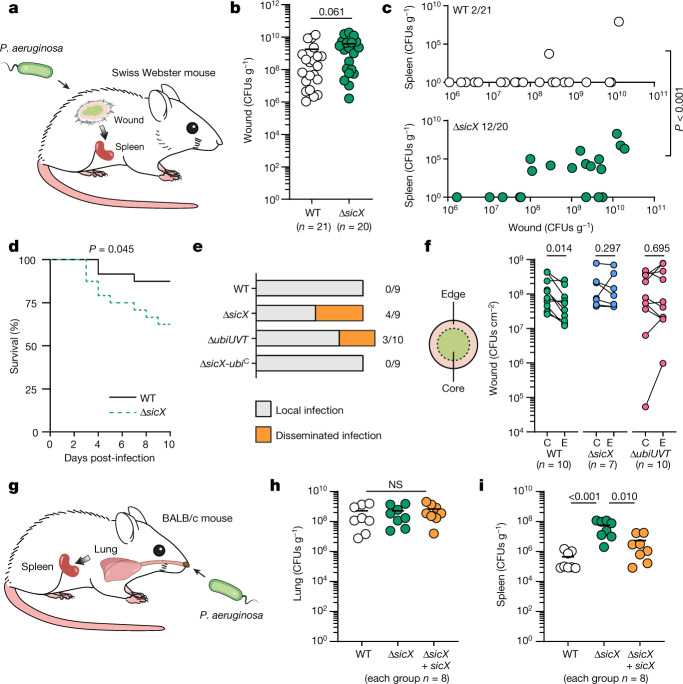
SicX governs chronic–acute lifestyle transition in the mammalian host. **a**, Schematic of the mouse chronic wound model (10-day infection). **b**, *P.* *aeruginosa* burden in wounds. Lines indicate means. **c**, *P.* *aeruginosa* burden in the spleen (*y* axis). Corresponding wound burden is indicated on the *x* axis. **d**, Survival curves of mice infected with WT (*n* = 24) and Δ*sicX* (*n* = 24). **e**, Restoration of *ubiUVT* translation in Δ*sicX* prevented dissemination, and deletion of *ubiUVT* in WT caused dissemination. **f**, *P.* *aeruginosa* spatial organization in wounds. C, core; E, edge. **g**, Schematic of the mouse pneumonia model (2-day infection). **h**, *P.* *aeruginosa* burden in lungs. Lines indicate means. **i**, *P.* *aeruginosa* burden in the spleen. Lines indicate means. Two-tailed Mann–Whitney test was used to compare the bacterial burden differences in **b**,**c**,**h**,**i**. Log-rank test and two-tailed Wilcoxon matched-pairs signed-rank test were used for **d**,**f**, respectively. Summary of *n* = 6 independent experiments for **b**–**d**; *n* = 3 independent experiments for **e**,**f**; *n* = 2 independent experiments for **h**,**i**. NS, not significant. Source Data

**Extended Data Fig. 5:**
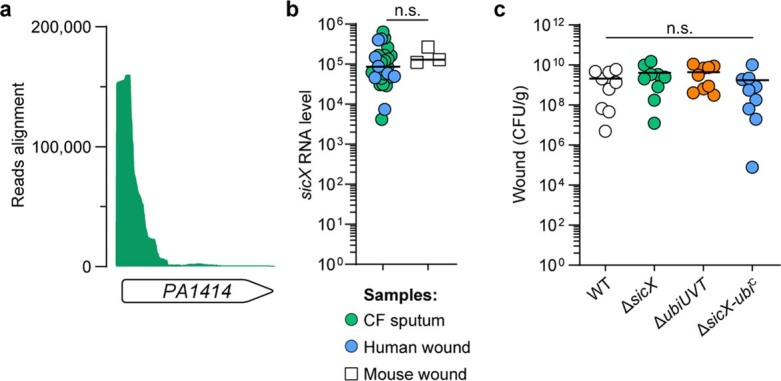
SicX expression in mouse chronic wounds. **a**. RNA-seq reads aligned to the *PA1414* locus (*sicX*) in mouse chronic wound infection. **b**. *sicX* RNA level in the mouse chronic wound is comparable to that in humans. Lines indicate means. Two-tailed Mann-Whitney test was used to compare the bacterial burdens (n.s., not significant). **c**. Bacterial burdens in the wound (dissemination information shown in Fig. 3e). Lines indicate means. *n* = 9 animals (for each group) over three independent experiments. Two-tailed Mann-Whitney test was used to compare the bacterial burdens of WT and each other mutant (n.s., not significant). Source Data

**Extended Data Fig. 6:**
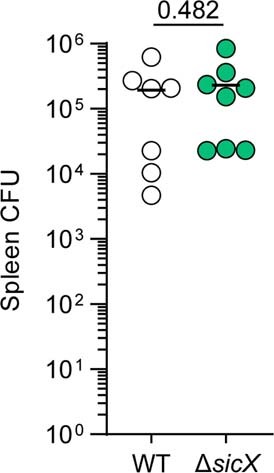
WT and Δ*sicX* showed similar spleen colonization in a systemic infection model. *P. aeruginosa* cells (~10^7^ CFU) were subcutaneously injected into the mouse inner thigh to induce systemic infection. Bacterial burdens in spleens were assessed at 16 h post-infection. WT (*n* = 7) and Δ*sicX* (*n* = 8). Lines indicate means. Two-tailed Mann-Whitney test was used. Source Data

Although we now have a more granular understanding of SicX function in vitro, how SicX affects *P.* *aeruginosa* pathogenesis remains unknown. Here we addressed this question by introducing WT *P.* *aeruginosa* and Δ*sicX* into a mouse chronic wound model (Fig. 3a). This model was chosen because it has been quantitatively shown to accurately recapitulate *P.* *aeruginosa* gene expression in human chronic infections^13^, and SicX sRNA in this model is expressed similarly to that in humans (Fig. 2a and Extended Data Fig. 5a,b). The model involves infection of surgically created full-thickness dorsal wounds^31^ (Fig. 3a). It is considered a chronic, non-lethal model because *P.* *aeruginosa* generally persists at the infection site for weeks without causing systemic dissemination. At 10 days post-infection, we assessed the bacterial burden in wound tissues as well as dissemination to the spleen. In wound tissues, the bacterial levels were similar between WT *P.* *aeruginosa* and Δ*sicX* (Fig. 3b), indicating that lacking SicX does not affect colonization. However, whereas only 2 of 21 WT-infected mice showed dissemination to the spleen, 12 of 20 Δ*sicX*-infected mice showed systemic dissemination (two-tailed Fisher’s exact test, *P* = 0.0009; Fig. 3c). In addition, Δ*sicX* caused higher lethality (Fig. 3d), which is not commonly observed with WT *P.* *aeruginosa* in this model. Dissemination generally occurred when the wound burden was high (>10^8^ colony-forming units (CFUs) g^−1^; Fig. 3c). However, among mice carrying a high wound burden (>10^8^ CFUs g^−1^), Δ*sicX* caused more systemic infection (12 out of 15) compared to WT (2 out of 11), indicating that dissemination did not solely depend on the wound burden (two-tailed Fisher’s exact test, *P* = 0.0043; Fig. 3c). Moreover, these observations are not due to differential fitness of WT and Δ*sicX* in spleens. This was demonstrated using an acute skin infection model in which systemic infection occurs shortly after subcutaneous injection into the mouse inner thigh. In this model, WT and Δ*sicX* exhibited similar colonization and growth in spleens (Extended Data Fig. 6). Taken together, our data indicate that SicX is a key player in *P.* *aeruginosa* chronic infection, the high expression of which promotes chronic localized infection.

**Extended Data Fig. 7:**
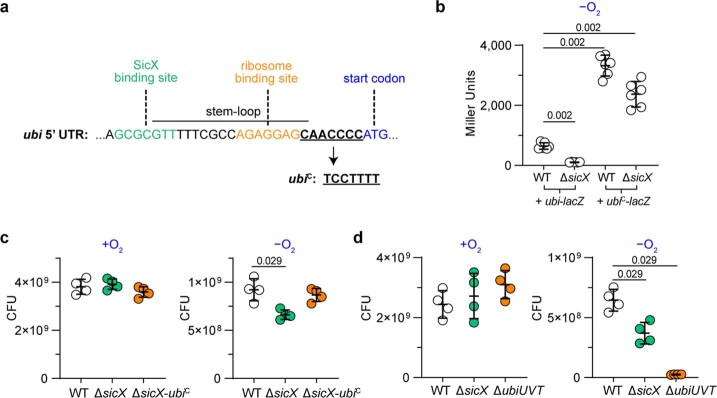
Decoupling the translation of *ubiUVT* from SicX. **a**. Schematic of mutagenesis in the 5’ UTR of *ubiUVT*: SicX binding site, ribosome binding site, and start codon are colored differently; the stem-loop structure is highlighted; mutagenesis (*ubi*^C^) is indicated with an arrow. **b**. Restoring *ubiUVT* translation in Δ*sicX*. β-galactosidase assay was performed to assess the translational activity of *ubiUVT* with and without 5’ UTR mutation as described in A (*n* = 6). **c–d**. Growth yield of colony biofilms in the presence or absence of oxygen (*n* = 4). Error bars represent standard deviation from the mean. Significant differences (compared to the WT) are indicated with asterisks (*, *P* < 0.05; **, *P* < 0.01; Two-tailed Mann-Whitney test).

To determine whether the increased dissemination and lethality of Δ*sicX* infection is linked to reduced anaerobic ubiquinone production, we first decoupled the translation of *ubiUVT* from SicX by creating a *P.* *aeruginosa* strain (Δ*sicX*-*ubi*^C^) that constitutively translates UbiUVT, even in the absence of SicX (Extended Data Fig. 7a,b). This was accomplished by introducing point mutations to the 5′ UTR of *ubiUVT* that probably alter its secondary structure and enable active translation. Δ*sicX*-*ubi*^C^ grew to similar yields to those of WT *P.* *aeruginosa* and significantly higher yields than those of Δ*sicX* under anaerobic conditions in vitro (Extended Data Fig. 7c), and this was due to the restoration of anaerobic ubiquinone synthesis (Fig. 2k). Next, using the mouse chronic wound model, we found that Δ*sicX*-*ubi*^C^ did not exhibit systemic dissemination (Fig. 3e). We also deleted the *ubiUVT* operon from WT *P.* *aeruginosa*, and the resulting strain did not grow anaerobically (Extended Data Fig. 7d) owing to the defect in anaerobic ubiquinone synthesis^23^. Notably, although all strains exhibited similar bacterial burdens in the wound (Extended Data Fig. 5c), systemic dissemination was observed only in Δ*sicX* and Δ*ubiUVT* infection (Fig. 3e), indicating that sufficient anaerobic ubiquinone synthesis contributes to chronic localized infection. Although the mechanism of how ubiquinone abundance influences infection lifestyles is unclear, we found that it affects the macroscale spatial distribution of *P.* *aeruginosa* cells in the wound: WT cells preferentially remained in the wound centre where the cells were originally inoculated, and Δ*sicX*-infected wounds contained equivalent cells in the centre and outer edges of the wounds (Fig. 3f). This is supported by our proteomic findings showing that Δ*sicX* exhibited increased levels of two proteins (encoded by *PA0223* and *PA0224*) that are biomarkers of dispersed *P.* *aeruginosa*^32^ (Supplementary Table 2). Moreover, Δ*ubiUVT* showed a similar spatial organization pattern to that of Δ*sicX* (Fig. 3f). Considering that the wound probably harbours oxygen gradients, we speculate that these different spatial distributions partly reflect varying oxygen availability across the wound. Collectively, these findings suggest that reduced anaerobic ubiquinone biosynthesis promotes the dissemination of Δ*sicX*.

**Extended Data Fig. 8:**
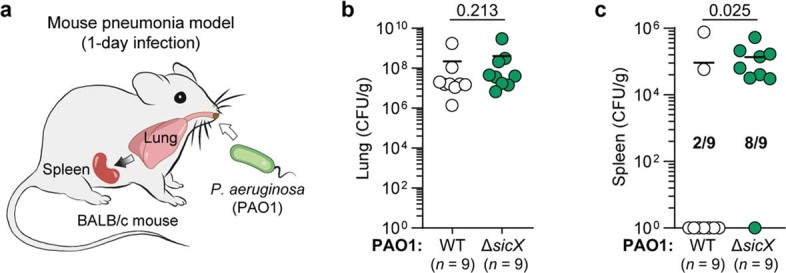
SicX (in PAO1 strain background) governs chronic-to-acute transition. **a**. Schematic of the mouse pneumonia infection experiment. **b**. *P. aeruginosa* burden in the lung. Lines indicate means. **c**. *P. aeruginosa* burden in the spleen. Lines indicate means. Two-tailed Mann-Whitney test was used to compare bacterial burdens. Summary of two biological replicates for **b** and **c**. Source Data

*P.* *aeruginosa* is notorious for causing a range of lung infections, and therefore, we also assessed the role of SicX in colonization and dissemination using a mouse pneumonia model (Fig. 3g). Here, *P.* *aeruginosa* establishes infection in the lung after intranasal administration, which in turn can lead to systemic dissemination depending on the virulence of individual strains. We found that mice infected with sublethal doses of WT and Δ*sicX* variants of PA14, a highly virulent strain, had similar bacterial burdens in the lungs at 48 h post-infection (Fig. 3h). By contrast, bacterial burdens in the spleens of Δ*sicX*-infected mice were approximately 100-fold higher than that of WT-infected mice, and complementation with the truncated *sicX* allele (depicted in Fig. 2g) in *trans* reduced dissemination (Fig. 3i), indicating that lacking SicX promotes systemic infection. Moreover, we carried out experiments using another strain, PAO1, that is less acutely virulent than PA14. We found that WT and Δ*sicX* exhibited similar colonization in the lungs, and whereas only two of nine WT-infected mice showed dissemination to the spleen, eight of nine Δ*sicX*-infected mice showed systemic dissemination (two-tailed Fisher’s exact test, *P* = 0.015; Extended Data Fig. 8). These observations indicate that SicX promotes chronic localized infection in multiple mammalian infection models.

## SicX is a biomarker of chronic–acute transition

**Fig. 4:**
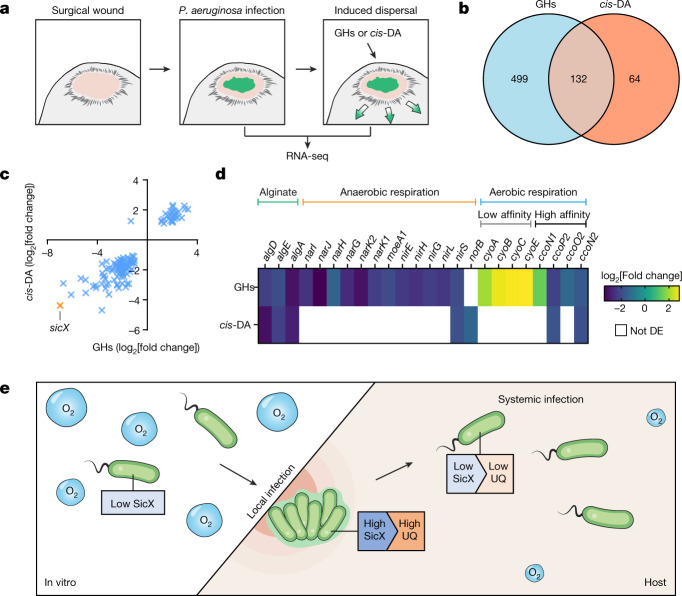
SicX is a biomarker for chronic–acute lifestyle transition. **a**, Schematics of in vivo dispersal of mature biofilms in mouse chronic wounds. **b**, Venn diagram of genes differentially expressed before (*n* = 3 animals) and after GH (*n* = 2) or *cis*-DA (*n* = 2) treatment. **c**, *sicX* (orange) is the top downregulated gene among the 132 differentially expressed genes identified under both GH and *cis*-DA treatments. **d**, Heat map of differentially expressed (DE) genes involved in alginate synthesis and secretion, anaerobic respiration and aerobic respiration. Aerobic terminal oxidases with low or high oxygen affinities are indicated. Blank cells indicate genes not identified as differentially expressed. **e**, A working model of how SicX allows *P.* *aeruginosa* to establish chronic local infection in a host environment with low oxygen. No or low SicX promotes systemic infection. Source Data

**Extended Data Fig. 9:**
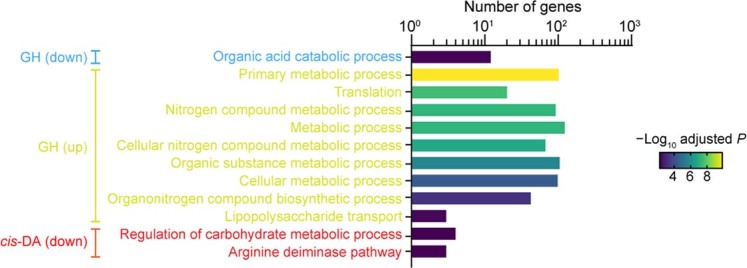
Functional enrichment of differentially-expressed genes in *P. aeruginosa* after GH or *cis*-DA treatment. Gene Ontology (GO) terms are labeled on the left. One-tailed Fisher’s exact test was performed. Down, downregulated; up, upregulated.

As the presence or absence of SicX dictates distinct infection lifestyles, we next considered whether SicX sRNA abundance changes during the transition between chronic and acute infections. To test this, we used the mouse chronic wound model in which WT *P.* *aeruginosa* was first allowed to establish local infection and then treated with glycoside hydrolases (GHs) or *cis*-2-decenoic acid (*cis*-DA) to induce systemic dissemination, mimicking the chronic-to-acute transition (Fig. 4a). GHs and *cis*-DA were used as they offer distinct mechanisms that drive biofilm dispersal: GHs target biofilm exopolysaccharides by hydrolysing the glycosidic linkages to passively induce dispersal^33–35^, whereas *cis*-DA is a fatty acid signalling molecule that actively drives dispersal by regulating bacterial gene expression and physiology^36–38^. Importantly, it has been shown that GH-induced biofilm dispersal in this model can cause lethal septicaemia^35^. Here we compared *P.* *aeruginosa* transcriptomes before (that is, mature biofilms in wound tissues) and after (that is, dispersed cells) the treatment with GHs or *cis*-DA (Fig. 4b). Functional enrichment analyses support the notion that GHs and *cis*-DA induced different physiological and metabolic changes during in vivo dispersal (Extended Data Fig. 9). Strikingly, among the genes that were differentially expressed in response to both treatments, we found that *sicX* was the top downregulated gene (Fig. 4c), supporting the notion that SicX levels change markedly during the chronic-to-acute transition. We also found that genes involved in alginate synthesis (*algA* and *algD*) and export (*algE*) were downregulated during biofilm dispersal (Fig. 4d). Moreover, the denitrification pathway (*nir*, *nar* and molybdopterin cofactor genes in response to GHs; *nir* and *nor* genes in response to *cis*-DA) and high-oxygen-affinity oxidases (*cbb*_3_-type oxidase in response to both GHs and *cis*-DA) were downregulated, whereas low-oxygen-affinity oxidases (Cyo oxidase in response to GHs) were upregulated (Fig. 4d), indicating an increase of oxygen availability during dispersal. Collectively, our observations identify *sicX* as a biomarker for chronic-to-acute transition and suggest oxygen as a key environmental signal during this process.

In conclusion, by leveraging bacterial transcriptomes from human infections, we discovered that *P.* *aeruginosa* produces an oxygen-responsive sRNA, SicX, the abundance of which orchestrates the transition between chronic and acute infections (Fig. 4e). The ability to respond to stressful environments is crucial for the survival of many organisms, during which sRNAs play key roles in fine-tuning gene expression. For example, in *Salmonella* infection, the sRNA PinT is induced and regulates gene expression to facilitate the transition from the invasion stage to intracellular survival^39^. In another case, *Helicobacter pylori* silences the expression of the sRNA HPnc4160 during infection, which upregulates target genes to promote bacterial colonization in the host and potentially increase the risk of gastric carcinogenesis^40^. In this study, we provide a compelling case of an sRNA directing important lifestyle choices during *P.* *aeruginosa* infection. Moreover, SicX shows promise as a prognostic and diagnostic biomarker, as it is the most highly responsive gene during the chronic-to-acute transition.

**Extended Data Fig. 10:**
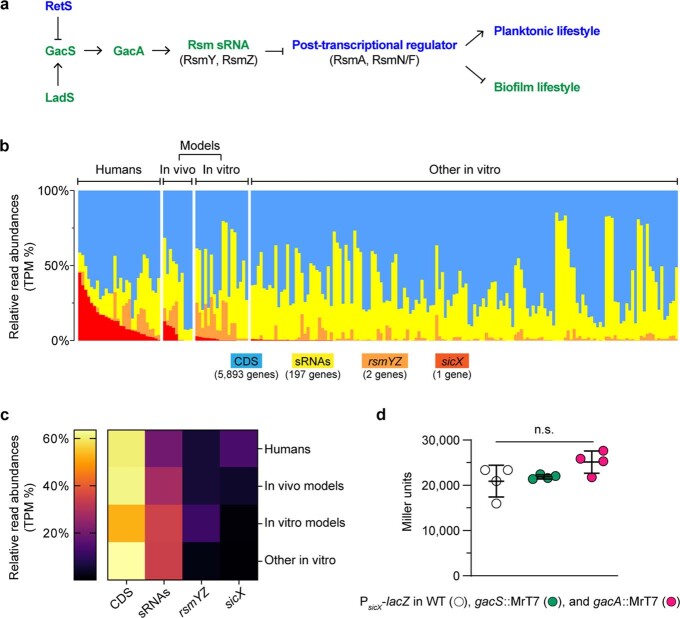
Relatively low expression of Rsm sRNAs during infections. **a**. Schematics of the Gac-Rsm cascade in *P. aeruginosa*. Components that promote biofilm (green) and planktonic (blue) lifestyles are color-coded. **b**. Relative transcript abundance (TPM, transcripts per million) of *sicX* compared to all protein-coding sequences (CDS), *rsmYZ* sRNAs, and the remaining non-coding sRNAs in 202 transcriptomes examined in Fig. 2a. Transcriptomes are sorted from the highest to the lowest *sicX* TPM. **c**. A heatmap showing the mean TPM of CDS, sRNAs, *rsmYZ*, and *sicX* in *P. aeruginosa* transcriptomes. **d**. β-galactosidase assays demonstrating the expression of *sicX* is independent from GacS-GacA. P_*sicX*_-*lacZ*, *lacZ* transcriptionally fused to *sicX* promoter. MrT7, MAR2xT7 transposon. *n* = 4 in all experiments. Error bars represent standard deviation from the mean. Significant difference (compared to WT) was analyzed with a Two-tailed Mann-Whitney test (n.s., not significant).

Although decades of in vitro studies have defined key regulatory systems governing the biofilm–planktonic transition in *P.* *aeruginosa*, robust evidence substantiating their roles in the mammalian host is lacking. For example, the Gac–Rsm signalling cascade is widespread in many bacterial species, and it is known to modulate the expression of features associated with planktonic or biofilm lifestyle in *P.* *aeruginosa*. This cascade relies on the interactions between global post-transcriptional regulators (RsmA or RsmN (also known as RsmF)) and Rsm sRNAs to fine-tune gene expression^6^ (Extended Data Fig. 10a). However, we found that Rsm sRNAs are expressed only at low levels in patients chronically infected with *P.* *aeruginosa* (Extended Data Fig. 10b,c), indicating that they are minimally involved in maintaining long-term infection. Next, although studies have shown that inactivation of specific components of Gac–Rsm led to fitness or colonization defects in acute infection models^7,41^, compelling evidence supporting a lifestyle switch model during mammalian infection remains lacking. Moreover, we found that the regulation of SicX expression is independent from the GacS–GacA two-component system (Extended Data Fig. 10d). In addition, the primary *P.* *aeruginosa* strain used in this study (PA14) has a mutation in *ladS*, a sensor kinase that stimulates GacS–GacA activity and promotes biofilm features^42^, yet this strain still established chronic localized infection in the mouse chronic wound model (Fig. 3b). Like many other pathogenic bacteria, *P.* *aeruginosa* colonizes a wide range of ecological niches including host-free ecosystems, and as a result, certain regulatory features controlling the biofilm–planktonic transition may have been selected under conditions that are unrelated to human pathogenesis. Our study thus provides a framework for discovering important pathogenic traits by leveraging bacterial gene expression information from human infections.

## Methods

### Media and growth conditions

*P.* *aeruginosa* cells were routinely cultured in brain heart infusion (BHI) broth at 37 °C with shaking (250 r.p.m.) unless otherwise specified. *Pseudomonas* isolation agar (PIA) was used for selecting *P.* *aeruginosa* against other microbes. *Escherichia coli* cells were cultured in lysogeny broth. To prepare plates, 1.5% (w/v) agar was used. Antibiotics were added to the medium as necessary: 60 µg ml^−1^ gentamicin, 75 µg ml^−1^ Tet or 300 µg ml^−1^ carbenicillin for *P.* *aeruginosa*; 10 µg ml^−1^ gentamicin, 10 µg ml^−1^ Tet or 100 µg ml^−1^ carbenicillin for *E.* *coli*. For *P.* *aeruginosa* static or anaerobic cultivation, 50 mM KNO_3_ was added to BHI broth. The anaerobic condition was maintained in a vinyl anaerobic chamber (Coy Lab Products) with the following atmosphere: 85% N_2_, 10% CO_2_ and 5% H_2_.

### Plasmids and bacterial strains

Strains, plasmids and primers used in this study are listed in Supplementary Table 3. To create transcriptional *lacZ* fusions (pPC100–102), desired promoter regions were PCR amplified (Q5, New England Biolabs) using PA14 genomic DNA as the template, and the resulting amplicons containing overlapping regions of about 25 base pairs (bp) were ligated into the mini-CTX-*lacZ* vector (linearized with EcoRI and KpnI) in Gibson Assembly Master Mix (New England Biolabs). To create *sicX* complementation plasmids (pPC103–106), the *sicX* promoter and *sicX* alleles (containing mutations as described in Supplementary Table 3) were ligated into mini-CTX1 vector (linearized with EcoRI and KpnI) through Gibson assembly. All *sicX* point mutations (pPC107–124) were constructed with the Q5 Site-Directed Mutagenesis Kit (New England Biolabs), using mini-CTX1-*sicX*^Truncate^ (pPC106) as the template. To create translational *lacZ* fusions (pPC125–126), the promoter region of *ubiU* was PCR amplified and then ligated into pSW205 between the EcoRI and BamHI sites. All *ubiUVT* 5′ UTR point mutations (pPC127–145) were constructed with the Q5 Site-Directed Mutagenesis Kit (New England Biolabs), using pSW205-P_*ubi*_-*lacZ* (pPC125) as the template. To construct plasmids used for markerless gene deletion (pPC146–147, 149–150) and mutagenesis (pPC148), upstream and downstream regions (about 1,000 bp) flanking the target gene or region were PCR amplified (containing mutations in the overlapping region if needed) and ligated into pEXG2 (linearized with SacI and KpnI) through Gibson assembly. All plasmids were verified by PCR, restriction enzyme digestion and, if necessary, DNA sequencing.

Verified plasmids were then transformed into *E.* *coli* SM10 λ*pir*, followed by conjugation into *P.* *aeruginosa* PA14, PAO1, B1 and their derivatives. The pSW205-derived plasmids were directly electroporated into *P.* *aeruginosa*. Appropriate antibiotics were used for selection as indicated in Supplementary Table 3. To create markerless in-frame deletion or mutagenesis, conjugants with the deletion cassette integrated at the target loci were counter-selected on low-salt lysogeny broth agar plates supplemented with 5% sucrose. The successful deletion was screened by PCR using flanking primers and verified by DNA sequencing if needed.

### Homologue identification and sequence analysis

Blastn searches were carried out on pseudomonas.com and the National Center for Biotechnology Information website to identify orthologues of *sicX* and *ubiUVT* across the *Pseudomonas* genus and other organisms. The *sicX* sequence (for Fig. 1c and Extended Data Fig. 1), *sicX* with its neighbouring genes (Fig. 1d) and *ubiUVT* with its upstream intergenic region (for Extended Data Fig. 4) from the *P.* *aeruginosa* PA14 strain were used as queries. Orthologues identified from complete bacterial genomes with *E* values of <1 × 10^−4^, percentage identity >90% and query coverage values of >90% were retained for further analysis. Sequences of *sicX* and *ubiUVT* orthologues were aligned using MUSCLE^43^. To analyse the sequence conservation of *sicX* and *ubiUVT* orthologues, we first aligned *sicX* and *ubiUVT* orthologues using MUSCLE. Next, alignment ends were trimmed and gaps were removed, and percentages of orthologue sequences that are identical to *sicX* and *ubiUVT* queries were calculated at each nucleotide position in R. To create Extended Data Fig. 4b,c, the alignments were visualized in Jalview^44^ using the default nucleotide colour scheme.

### β-galactosidase assay

*P.* *aeruginosa* cells were grown overnight in BHI broth supplemented with 50 mM KNO_3_. The overnight culture was diluted to an optical density at 600 nm (OD_600nm_) of about 0.05 in fresh BHI broth (with 50 mM KNO_3_) and then grown to the log phase (OD_600nm_ around 0.5). Log-phase cells were diluted to OD_600nm_ 0.2 and transferred to the anaerobic chamber for 3-h shaking (at 100 r.p.m.) cultivation at 37 °C. A 100 μl volume of the anaerobic culture was used to conduct the β-galactosidase assay (as described in ref. ^45^). Aerobic log-phase cultures were used as comparisons. For static cultivation, log-phase cultures were diluted to OD_600nm_ 0.05 and incubated in a 96-well plate (500 μl in each 1-ml-deep well) statically at 37 °C for 3 h. The static cultures were then used for the β-galactosidase assay.

For making Extended Data Fig. 3a,b, strains harbouring *ubiUVT*-*lacZ* translational fusions were spotted (3 μl of OD_600nm_ 0.1 cultures) onto 0.2-μm Nuclepore track-etched polycarbonate membranes (Whatman) that were placed (rough side facing upwards) on BHI agar plates containing 200 µg ml^−1^ X-gal, 50 mM KNO_3_ and 100 µg ml^−1^ carbenicillin. The plates were then incubated in the anaerobic chamber (protected from light) at 37 °C for 20 h before imaging. β-galactosidase activities were estimated as described previously^46^. Briefly, images of colony biofilms were converted to 8 bit in ImageJ. Thresholding was carried out to define colony objects for measurements. Mean pixel intensity was measured for each colony and subtracted with the mean pixel intensity of a colony of WT PA14.

### Northern blot

*P.* *aeruginosa* cells were grown aerobically and anaerobically for total RNA extraction. To prepare aerobic cultures, log-phase cells were diluted to OD_600nm_ 0.01 in BHI (with 50 mM KNO_3_) and incubated at 37 °C with shaking for 3 h. To prepare anaerobic cultures, log-phase cells were diluted to OD_600nm_ 0.1 in BHI (with 50 mM KNO_3_) and incubated in the anaerobic chamber for 4 h with shaking at 37 °C. For total RNA extraction, *P.* *aeruginosa* cultures were first stored in RNAlater at 4 °C overnight. Next, cells were pelleted and resuspended in RNase-free TE buffer containing 1 mg ml^−1^ lysozyme. Samples were incubated at 37 °C for 30 min to enzymatically lyse cells. A 1 ml volume of RNA-Bee was then added, and cells were further mechanically lysed by bead beating three times for 30 s. A 200 μl volume of chloroform was added to the samples, and the aqueous and organic phases were separated by centrifugation at 13,000*g* for 30 min at 4 °C. The aqueous phase that contained RNA was mixed with 0.5 ml isopropanol and 20 μg linear acrylamide. After overnight incubation at −80 °C, RNA was pelleted and washed with 75% ethanol. Air-dried pellets were resuspended in 100 μl of RNase-free water. The RNA concentration for each sample was determined with a NanoDrop spectrophotometer (Thermo Fisher Scientific). Approximately 1 μg of total RNA was separated on a 15% polyacrylamide gel electrophoresis–urea gel and transferred to Hybond-N+ membranes (GE Healthcare). The membranes were then crosslinked with an ultraviolet crosslinker (UVP) and probed in hybridization solution (Takara) with the indicated DNA oligonucleotides (listed in Supplementary Table 3) that were radiolabelled with [γ-^32^P]ATP (Perkin Elmer) by T4 polynucleotide kinase (New England Biolabs). Probed membranes were exposed to a phosphor screen and visualized using a Typhoon Biomolecular Imager (GE Healthcare). When necessary, membranes were stripped by incubating them with boiled 0.1% SDS with agitation for 15 min three times. The sizes of RNA were estimated with the RiboRuler Low Range RNA Ladder (Thermo Fisher Scientific) as well as the migration of xylene cyanol (about 28 nt) and bromophenol blue (about 8 nt).

### Colony biofilm assay

Bacterial cultures in BHI (with 50 mM KNO_3_) were grown to the log phase (OD_600nm_ around 0.5) and then diluted to OD_600nm_ 0.02 with fresh medium. Next, 10 μl diluted cultures were spotted onto 0.2-μm Nuclepore track-etched polycarbonate membranes (Whatman) that were placed (smooth side facing upwards) on BHI agar plates (with 50 mM KNO_3_). The plates were then incubated in the anaerobic chamber at 37 °C for 16 h. Membranes containing *P.* *aeruginosa* biofilms were transferred to the BeadBug prefilled tubes that contained 1 ml phosphate-buffered saline (PBS). Biofilms were mechanically disrupted by bead beating for 45 s. CFUs were determined by spreading the diluted cell suspensions on PIA plates.

### Quantitative proteomics and data analysis

*P.* *aeruginosa* PA14 and Δ*sicX* cells were collected under both static and anaerobic growth conditions as described above (in the section entitled β-galactosidase assay).

For static growth condition, three biological replicates for each strain were collected. Cell pellets were resuspended in lysis buffer (100 mM Tris-HCl, 50 mM NaCl, 10% (v/v) glycerol, 1 mM Tris(2-carboxyethyl)phosphine) (TCEP), pH 7.5) with cOmplete Mini protease inhibitor cocktail (Roche). Following three rounds of sonication (3 × 10 s) on ice, supernatants were clarified by sedimentation (21,130*g*, 15 min, 4 °C). Aliquots (100 μg) of each sample were reduced with TCEP, alkylated with iodoacetamide and labelled with tandem mass tags (TMTs). TMT labelling was carried out according to the protocol specified by the manufacturer (Thermo Fisher).

Liquid chromatography–tandem mass spectrometry (LC–MS/MS) experiments were carried out using a Dionex Ultimate 3000 rapid separation LC (RSLC) nano-ultraperformance LC system (Thermo Fisher Scientific) and a Lumos Orbitrap mass spectrometer (Thermo Fisher Scientific). Separation of peptides was carried out by C18 reversed-phase chromatography at a flow rate of 300 nl min^−1^ using a Thermo Scientific reversed-phase nano EASY-spray column (Thermo Scientific PepMap C18; 2-mm particle size, 100-Å pore size, 75-mm i.d. by 50-cm length). Solvent A was water/0.1% formic acid, and solvent B was 80% (v/v) acetonitrile/20% water/0.1% formic acid. The linear gradient used was 2% to 40% solvent B over 93 min (the total LC run time was 120 min, including a high-organic-wash step and column re-equilibration).

The eluted peptides were sprayed into the mass spectrometer by means of an EASY-Spray source (Thermo Fisher Scientific). All *m*/*z* values representing eluting peptide ions were measured in an Orbitrap mass analyser (set at a resolution of 120,000) and were scanned at between *m*/*z* 380 and 1,500 Da. Data-dependent MS/MS scans (top speed) were used to automatically isolate and fragment precursor ions by collision-induced dissociation (normalized collision energy value, 35%) analysed in the linear ion trap. Singly charged ions and ions with unassigned charge states were excluded from selection for MS/MS, and a dynamic exclusion window of 70 s was used. The top 10 most abundant fragment ions from each MS/MS event were then selected for a further stage of fragmentation by synchronous precursor selection and MS/MS/MS^47^ in the high-energy-collision cell using high-energy collisional dissociation (normalized collision energy value of 65%). The *m*/*z* values and relative abundances of each reporter ion and of all fragments (mass range, 100 to 500 Da) in each MS3 step were measured in the Orbitrap analyser, which was set at a resolution of 60,000. This was carried out in cycles of 10 MS3 events, after which the Lumos instrument reverted to scanning the *m*/*z* ratios of the intact peptide ions and the cycle continued.

Proteome Discoverer v2.1 (Thermo Fisher Scientific) and Mascot (Matrix Science) v2.6 were used to process raw data files. Data were aligned to the sequences of *P.* *aeruginosa* UCBPP-PA14 (with common repository of adventitious proteins (cRAP) v1.0). The R package MSnbase v2.13 (ref. ^48^) was used for processing proteomics data. Protein differential abundances were evaluated using the Limma package v3.44.3 (ref. ^49^). Differences in protein abundances were statistically determined using Student’s *t*-test with variances moderated by the use of Limma’s empirical Bayes method. *P* values were adjusted for multiple testing by the Benjamini–Hochberg method^50^. Proteins were considered to have increased or decreased in abundance only when their log_2_[fold change] value was greater than 1 or less than −1, respectively, and when their *P* value was <0.01.

For anaerobic growth condition, two biological replicates for PA14 and three biological replicates for Δ*sicX* were collected as described above (in the section entitled β-galactosidase assay). Briefly, the proteins in each sample were reduced, alkylated and digested with trypsin according to the filter-aided sample preparation protocol^51^. The peptides were labelled with TMTs, separated by high-pH reversed-phase chromatography as described previously^52^. They were pooled into 12 fractions as described previously^53^. Each fraction was analysed by nano-LC–MS/MS, and peptides were identified as previously described^54^ with the following modifications. Reversed-phase chromatography was carried out using an in-house packed column (40 cm long × 75 μm inner diameter × 360 μm outer diameter, Dr. Maisch GmbH ReproSil-Pur 120 C18-AQ 1.9-µm beads) and a 120-min gradient. The Raw files were searched using the Mascot algorithm (v2.5.1) against a protein database constructed by combining the sequences of *P.* *aeruginosa* UCBPP-PA14 and a contaminant database (cRAP, downloaded 21 November 2016 from http://www.thegpm.org) through Proteome Discoverer v2.1. Only peptide spectral matches with expectation value of less than 0.01 (‘high confidence’) were used for our analyses as described above.

### Lipid extractions and ubiquinone (UQ9) quantification

To prepare cells for lipid extractions, we first diluted log-phase cultures (OD_600nm_ 0.5; grown aerobically) into the anaerobic BHI broth (supplemented with 50 mM KNO_3_) to a calculated OD_600nm_ 0.05, and after 6-h anaerobic incubation with shaking, cells were collected, washed once with PBS and flash-frozen in liquid nitrogen. Cell numbers were estimated with OD_600nm_.

Frozen samples were thawed on ice. A 100 µl volume of ice-cold isopropanol with 10 nM coenzyme Q10-d_9_ (deuterated UQ10 as internal standard; Sigma-Aldrich) was added to 10^9^ cells. With the addition of glass beads, samples were vortexed briefly and homogenized by TissueLyzer for 5 min twice, followed by centrifugation at 21,100*g* for 5 min. After centrifugation, supernatant was diluted tenfold and transferred to 4 °C. Ultraperformance LC–MS was carried out using an UltiMate 3000 fitted with an Accucore C30 column (2.1 × 150 mm, 2.6 µm particle size; Thermo Fisher) and coupled to an Orbitrap ID-X mass spectrometry system.

The chromatographic method for sample analysis involved elution with acetonitrile/water (60:40, v/v) with 10 mM ammonium formate and 0.1% formic acid (mobile phase A) and isopropanol/acetonitrile (90:10, v/v) with 10 mM ammonium formate and 0.1% formic acid (mobile phase B) at 0.4 ml min^−1^ flow rate using the following gradient programme: 0 min 20% B; 1 min 60% B; 5 min 70% B; 5.5 min 85% B; 8 min 90% B; 8.2 min 100% B hold to 10.5 min, then 10.7 min 20% B hold to 12 min. The column temperature was set to 50 °C, and the injection volume was 5 µl.

The targeted molecules UQ9 and deuterated UQ10 were fragmented by high-energy collisional dissociation at 20 collision energy in positive mode. MS/MS transitions for UQ9 and deuterated UQ10 are 812.66/197.08 and 889.77/206.18. Standard curves for UQ9 and deuterated UQ10 were generated. The amounts of UQ9 and deuterated UQ10 in the samples were calculated on the basis of the standard curve.

### Planktonic growth and competition assays

For generating growth curves, bacterial cells in BHI (with 50 mM KNO_3_) were first grown aerobically to the log phase (OD_600nm_ around 0.5) and then diluted to OD_600nm_ 0.01 with either aerobic or anaerobic BHI (with 50 mM KNO_3_) broth. Aerobic and anaerobic growth of *P.* *aeruginosa* at 37 °C (with shaking) were monitored by measuring the OD_600nm_.

For long-term competition experiments, PA14, Δ*sicX*, Tet^R^ (resistance provided by mini-CTX1) PA14 and Tet^R^ Δ*sicX* were grown to the log phase (OD_600nm_ around 0.5) in BHI (with 50 mM KNO_3_) before diluting to OD_600nm_ 0.4 with fresh medium. PA14 and Tet^R^ Δ*sicX* were mixed at 1:1 ratio, and 100 μl of cell mixtures was transferred to 4 ml anaerobic BHI (with 50 mM KNO_3_), followed with shaking incubation at 37 °C under anaerobic conditions. After 24 h, 20 μl of the culture was transferred to another tube containing 4 ml anaerobic BHI (with 50 mM KNO_3_). The culture was then consecutively passaged daily in a similar fashion. The competition between Tet^R^ PA14 and Δ*sicX* was conducted similarly to that described above. CFU values of Tet^R^ and Tet^S^ cells were estimated by plating cultures (on each day) on PIA as well as PIA containing 75 µg ml^−1^ Tet.

### In silico analyses of RNA folding and RNA–RNA interaction

RNA secondary structures were predicted using mfold^26^ with default parameters. IntaRNA 2.0 was used to predict base-pairing interactions between SicX sRNA and *ubiUVT* mRNA^25^ with default settings, and the minimum number of base pairs in the seed region was set as 7.

### Mouse chronic wound model

Mouse chronic wound infections were carried out with female Swiss Webster mice (8–10 weeks old) essentially as described previously^55^ with a few modifications. Briefly, dorsal full-thickness skin excision was carried out in which a small circular wound (with a diameter of 1.5 cm) was surgically administered. After excision, the wound was immediately covered with a semipermeable polyurethane dressing. A total of 10^4^ CFUs of *P.* *aeruginosa* cells were injected onto the wound bed underneath the dressing to establish infection. As bandages are required to reduce the contractile healing and minimize wound contamination with other bacteria, they were closely monitored and replaced when necessary throughout the infection course.

For assessing the bacterial burdens of WT and Δ*sicX* infection, wound tissues and spleens were collected at 10 days post-infection or at an early time point if the animal was found moribund (moribund mice that were not identified immediately were excluded from the CFU analysis). Tissues were homogenized in PBS for 45 s in BeadBug tubes with 2.8-mm steel beads (Sigma-Aldrich) using a Mini-Beadbeater-16 (BioSpec Products). To enumerate CFUs, homogenized samples were serially diluted and plated on PIA plates. For studying the dissemination outcomes of WT, Δ*sicX*, Δ*ubiUVT* and Δ*sicX*-*ubi*^C^ infection (Fig. 3e), at 10 days post-infection, distal organs including spleens, livers and gallbladders were collected for assessing the presence or absence of *P.* *aeruginosa* cells. For studying the macroscale organization of *P.* *aeruginosa* infection, the wound was excised at 4 days post-infection, and a biopsy punch of 10 mm diameter (Acu-Punch, Thermo Fisher Scientific) was used to separate the edge (about 0.98 cm^2^) and core (about 0.77 cm^2^) regions. The CFUs were then normalized by the surface area for comparison purposes.

### In vivo biofilm dispersal

In vivo biofilm dispersal was carried out with female Swiss Webster mice (8–10 weeks old) essentially as described previously^31^ with a few modifications. Briefly, the mouse surgical excision wound was administered as demonstrated above. A total of 10^4^ CFUs of *P.* *aeruginosa* cells (PAO1) were injected onto the wound bed underneath the dressing to establish infection. At 48 h post-infection (after the formation of mature biofilms in wounds), the established wound infections were treated through topical application of GHs or *cis*-DA to induce systemic dissemination. Specifically, GHs are composed of bacterial α-amylase (from *Bacillus subtilis*; MP Biomedicals) and fungal cellulase (from *Aspergillus niger*; MP Biomedicals). A 10% (w/v) α-amylase and cellulase (in a 1:1 combination) solution was prepared by dissolving powdered enzymes in PBS. *cis*-DA (Cayman Chemical Company) was diluted in PBS to a final concentration of 500 nM. GH and *cis*-DA solutions were prepared immediately before use. Wound beds were irrigated with a GH or *cis*-DA solution in three separate topical infusions with 30 min of dwell time for each. Topical infusions containing dispersed cells (*n* = 2 animals for each treatment) were aspirated and immediately placed in RNAlater (Thermo Fisher Scientific). In separate animals, *P.* *aeruginosa*-infected wound tissues (*n* = 3 animals) were carefully excised from the surrounding uninfected tissue and the underneath muscle layer at 48 h post-infection (without GH or *cis*-DA treatment) as a comparison. Infected wound tissues were immediately placed in RNAlater (Thermo Fisher Scientific).

### Mouse pneumonia model

Mouse pneumonia infection was carried out with female BALB/c mice (6–8 weeks old) essentially as described previously^56^ with a few modifications. Briefly, mice were anaesthetized by intraperitoneal injection of 0.2 ml of a cocktail of ketamine (25 mg ml^−1^) and xylazine (12 mg ml^−1^). For infection with PA14-derived strains, mice were intranasally instilled with approximately 10^7^ CFUs (a sublethal dose) *P.* *aeruginosa* cells (in 25 μl PBS). Mice were euthanized at 48 h post-infection. Whole lungs and spleens were collected aseptically, weighed and homogenized in 1 ml of PBS. Tissue homogenates were serially diluted and plated on PIA for CFU enumeration. For infection with PAO1-derived strains, 5 × 10^7^ CFUs (a lethal dose) of *P.* *aeruginosa* cells were used as inocula, and tissues were collected at 24 h post-infection.

### Mouse subcutaneous infection model

Mouse subcutaneous skin infection (also known as the mouse abscess model) was carried out with female Swiss Webster mice (8 weeks old) essentially as described previously^55^ with a few modifications. Briefly, the mouse inner thigh was shaved and any remaining hair was removed with a depilatory agent (Nair). A 100 μl volume of approximately 1 × 10^7^ CFUs of *P.* *aeruginosa* cells (PA14 or Δ*sicX*) were subcutaneously injected to the inner thigh. Mice were euthanized at 16 h post-infection. Spleens were collected aseptically and homogenized in 1 ml PBS. Tissue homogenates were serially diluted and plated on PIA for CFU enumeration.

### RNA-seq library preparation

RNA extractions were carried out essentially as described previously^14^. For in vitro static cultivation of PA14, cells were first grown as described above (in the section entitled β-galactosidase assay) and immediately mixed with five volumes of RNAlater. After storage at 4 °C overnight, cells were pelleted, resuspended in nuclease-free TE buffer (Acros Organics) containing 1 mg ml^−1^ lysozyme, and transferred to bead-beating tubes containing a mixture of large and small beads (2-mm zirconia and 0.1-mm zirconia/silica, respectively). For in vivo biofilm dispersal experiments (as described above), infected tissues were removed from RNAlater, mixed with TE buffer, and transferred to bead-beating tubes. In vitro as well as tissue samples in TE were briefly disrupted by homogenization using a Mini-Beadbeater-16. Samples were then incubated at 37 °C for 30 min to enzymatically lyse cells. Next, 1 ml RNA-Bee was added to 300-μl sample homogenate, and samples were further mechanically lysed by bead beating three times for 30 s. A 200 μl volume of chloroform per 1 ml RNA-Bee was added, and the tubes were shaken vigorously for 30 s and then incubated on ice for 5 min. The samples were then centrifuged at 13,000*g* for 30 min at 4 °C to separate the aqueous and organic phases. The aqueous phase was transferred to a new microcentrifuge tube to which 0.5 ml isopropanol (per 1 ml RNA-Bee) and 20 μg linear acrylamide were added. After overnight incubation at −80 °C, samples were thawed on ice and centrifuged at 13,000*g* for 30 min at 4 °C. Pellets were washed twice with 1 ml of 75% ethanol, air dried for 5 min and resuspended in 100 μl of RNase-free water. The RNA concentration for each sample was determined with a NanoDrop spectrophotometer (Thermo Fisher Scientific). rRNA was depleted using a MICROBExpress Kit (Thermo Fisher Scientific) for the in vitro samples and a QIAseq FastSelect Kit (QIAGEN) for mouse-derived samples, followed by ethanol precipitation. The depleted RNA was fragmented for 2 min with the NEBNext Magnesium RNA fragmentation module kit, and cDNA libraries were prepared using the NEBNext multiplex small RNA library prep kit (New England Biolabs) according to the manufacturer’s instructions. Libraries were sequenced at the Molecular Evolution Core at the Georgia Institute of Technology on an Illumina NextSeq500 using 75-bp single-end runs.

### Analysis of RNA-seq datasets

In addition to the above-mentioned RNA-seq libraries prepared in this study, 202 publicly available *P.* *aeruginosa* RNA-seq datasets were analysed, including 28 *P.* *aeruginosa* transcriptomes derived from human samples, 28 from infection models and 146 from a range of well-defined in vitro conditions (listed in Supplementary Table 1). The quality of the raw sequencing reads was first confirmed with FastQC v0.11.7 (ref. ^57^). Next, RNA-seq reads were trimmed on the 3′ ends to remove the Illumina adaptor (AGA TCG GAA GAG CAC ACG TCT GAA CTC CAG TCA C) using Cutadapt 3.0 (ref. ^58^) with a minimum read length threshold of 22 bases. Trimmed reads were then mapped to the *P.* *aeruginosa* PA14 reference genome (available for download from pseudomonas.com) using Bowtie2 v2.4.2 with default parameters for end-to-end alignment^59^. As *PA1414* (*sicX*) was originally annotated as a protein-coding gene, we first assigned reads to protein-coding genes with featureCounts Subread v2.0.1 (ref. ^60^; results shown in Fig. 1a). For the remaining analyses (Figs. 1b, 2a and 4b–d and Extended Data Figs. 2b and 10), reads assigned to both protein-coding genes as well as 199 sRNAs^15^ were tallied.

Differential expression was determined with DESeq2 (Figs. 1a and 4b–d; ref. ^61^). To compare *sicX* expression levels in different samples (Fig. 1a and Extended Data Fig. 5), raw reads were normalized using the varianceStabilizingTransformation() function in the DESeq2 package. To estimate the relative transcript abundance of various features (protein-coding genes, sRNAs, *sicX* and/or *rsmYZ*), we calculated TPM. First, raw counts were normalized by feature (gene) length to generate reads per kilobase (RPK). Next, all RPK values were counted up and divided by 1,000,000 to determine the scaling factor. Finally, RPK of each gene was divided by the scaling factor. The resulting TPM values were used for making Figs. 1b and 2a and Extended Data Figs. 2b and 10. To examine the alignment pattern of *sicX* reads, samtools v1.13 was used to measure the read depth encompassing the *sicX* locus at each nucleotide position^62^. The read depth was then normalized using the estimateSizeFactors() function in DESeq2 before plotting.

### Phylogenetic analysis

A maximum-likelihood phylogenetic tree was constructed in PhyML 3.0 (ref. ^63^) using 365 16S rRNA sequences representing diverse *Pseudomonas* isolates. For better visualization of the tree, we included only approximately 50 16S sequences from the *P.* *aeruginosa* strains. The 16S sequences were aligned with MUSCLE and used as an input in PhyML 3.0. The best-fitting evolutionary models were predicted with the corrected Akaike information criterion. The tree was visualized and annotated in iTOL^64^.

### Functional enrichment analysis

Gene Ontology enrichment analysis was conducted using Galaxy v2.0.1. Differentially expressed genes in response to GH or *cis*-DA treatment were analysed using the Gene Ontology file (obtained from http://geneontology.org/docs/download-ontology) and the *P.* *aeruginosa* PAO1 Gene Ontology term annotations (obtained from https://pseudomonas.com/goterms/list). A Benjamini–Hochberg test with *P*-value cutoff < 0.05 was carried out.

### Statistics and reproducibility

All experiments were carried out independently at least three times (unless otherwise stated in the figure legends) with similar observations. The exact *n* values for animal studies as well as in vitro experiments are provided in the figures and legends. Statistical analyses were conducted using Prism GraphPad 9 and are specified in the figure legends. For northern blotting, each experiment was conducted at least twice independently (by using freshly extracted RNA samples) with similar observations, and representative images are shown.

### Ethics statement

All mouse procedures were carried out in strict accordance with the recommendations in the *Guide for the Care and Use of Laboratory Animals* of the National Institutes of Health. All animals were treated humanely and housed in temperature- and humidity-controlled facilities (18–22 °C; 40–50%) with 12 h light–dark cycles. The protocol for the mouse chronic wound infection was approved by the Institutional Animal Care and Use Committee (IACUC) of Texas Tech University Health Sciences Center (protocol number 07044) and by the IACUC of Georgia Institute of Technology (protocol number A100127). The protocol for the mouse subcutaneous infection model was approved by the IACUC of Georgia Institute of Technology (protocol number A100127). For the mouse pneumonia infection model, all experimental procedures were conducted according to the guidelines of the Emory University IACUC, under approved protocol number DAR-201700441.

### Reporting summary

Further information on research design is available in the Nature Portfolio Reporting Summary linked to this article.

## Online content

Any methods, additional references, Nature Portfolio reporting summaries, source data, extended data, supplementary information, acknowledgements, peer review information; details of author contributions and competing interests; and statements of data and code availability are available at 10.1038/s41586-023-06111-7.

## Supplementary information

Supplementary Information This file contains Supplementary Fig. 1, Tables 1–3 and References.

Reporting Summary

## Data Availability

The proteomics datasets generated in this study have been deposited in the ProteomeXchange Consortium (http://proteomecentral.proteomexchange.org/cgi/GetDataset), with the accession numbers are PXD032985 and PXD032986. Raw reads of our RNA-seq studies have been deposited in the Sequence Read Archive (https://www.ncbi.nlm.nih.gov/sra) under BioProject numbers PRJNA934328 and PRJNA904394. The 202 publicly available *P.* *aeruginosa* transcriptomes analysed in this study are summarized in Supplementary Table 1, and can be obtained from the Sequence Read Archive (https://www.ncbi.nlm.nih.gov/sra) with the corresponding accession number. The *P.* *aeruginosa* PA14 reference genome (BioSample accession SAMN02603591) is available for download from https://pseudomonas.com. Source data are provided with this paper.

## Source data

Source Data Fig. 1
Source Data Fig. 2
Source Data Fig. 3
Source Data Fig. 4
Source Data Extended Data Fig. 5
Source Data Extended Data Fig. 6
Source Data Extended Data Fig. 8
