# Supplementary material for: A Pseudomonas aeruginosa small RNA regulates chronic and acute infection
Source: Nature. 2023 May 24;618(7964):358–64. doi: 10.1038/s41586-023-06111-7 (PMC10247376; doi:10.1038/s41586-023-06111-7)
Supplement: Supplementary file 1 — This file contains Supplementary Fig. 1, Tables 1–3 and References. [file 41586_2023_6111_MOESM1_ESM.pdf]

nature portfolio

https://doi.org/10.1038/s41586-023-06111-7

Supplementary information

A *Pseudomonas aeruginosa* small RNA regulates chronic and acute infection

In the format provided by the authors and unedited

Supplementary Figure 1. Source images of Northern blots reported in this study.

Fig. 2c

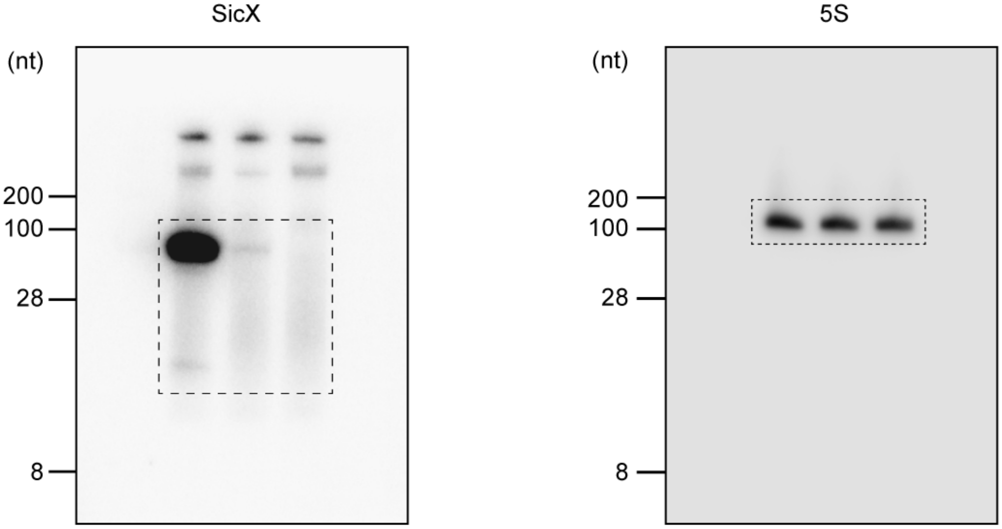

Extended data Fig. 2f

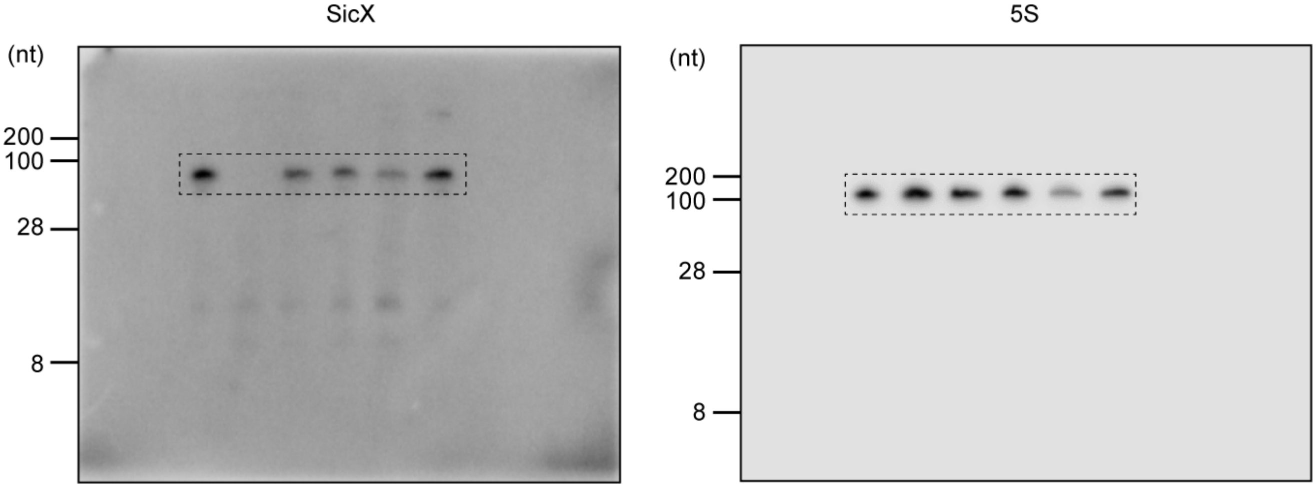

Notes:

(1) For each experiment, samples were run on the same gel. SicX and 5S rRNA (internal loading control) were probed separately.

(2) The sizes of RNA (nucleotides, nt) were estimated with the RiboRuler Low Range RNA Ladder (Thermo Fisher Scientific) as well as the migration of xylene cyanol (~28 nt) and bromophenol blue (~8 nt).

Supplementary Table 1. 202 *P. aeruginosa* transcriptomes analyzed in this study.

Accession*	Description	Type	Used in Fig.
SRR10267771	CF Sputum 54	Human	1AB, 2A, S10
SRR10267773	CF Sputum 49	Human	1AB, 2A, S10
SRR10267765	CF Sputum 24	Human	1AB, 2A, S10
SRR10267759	CF Sputum 43	Human	1AB, 2A, S10
SRR10267766	CF Sputum 13	Human	1AB, 2A, S10
SRR10267760	CF Sputum 37	Human	1AB, 2A, S10
SRR10267761	CF Sputum 36	Human	1AB, 2A, S10
SRR10267762	CF Sputum 35	Human	1AB, 2A, S10
SRR10267763	CF Sputum 33	Human	1AB, 2A, S10
SRR10267764	CF Sputum 30	Human	1AB, 2A, S10
SRR10267774	CF Sputum 48	Human	1AB, 2A, S10
SRR6833344	CF Sputum C	Human	1AB, 2A, S10
SRR6833347	CF Sputum D	Human	1AB, 2A, S10
SRR10267772	CF Sputum 53	Human	1AB, 2A, S10
SRR10267770	CF Sputum 6	Human	1AB, 2A, S10
SRR6833345	CF Sputum B	Human	1AB, 2A, S10
SRR6833351	CF Sputum E	Human	1AB, 2A, S10
SRR6833346	CF Sputum A	Human	1AB, 2A, S10
SRR6833349	CF Sputum G	Human	1AB, 2A, S10
SRR6833350	CF Sputum F	Human	1AB, 2A, S10
SRR6833323	Chronic wound 18	Human	1AB, 2A, S10
SRR6833342	Burn wound 2-3	Human	1AB, 2A, S10
SRR6833341	Burn wound 2-4	Human	1AB, 2A, S10
SRR6833348	Chronic wound 1	Human	1AB, 2A, S10
SRR6833325	Chronic wound 21	Human	1AB, 2A, S10
SRR6833324	Chronic wound 16	Human	1AB, 2A, S10
SRR6833340	Chronic wound 15	Human	1AB, 2A, S10
SRR6833343	Chronic wound 2	Human	1AB, 2A, S10
SRR10267769	Mouse chronic wound	Model	S5, S10
SRR1047593	Mouse chronic wound	Model	S5, S10
SRR1047595	Mouse chronic wound	Model	S5, S10
SRR1047581	Mouse burn wound	Model	S5, S10
SRR1047580	Mouse burn wound	Model	S5, S10
SRR6833316	SCFM2	Model	2A, S10
SRR6833322	SCFM2	Model	2A, S10
SRR6833331	SCFM2	Model	2A, S10
SRR6833330	SCFM2	Model	2A, S10
SRR6833326	SCFM2	Model	2A, S10
SRR6833327	SCFM2	Model	2A, S10
SRR6833328	SCFM2	Model	2A, S10
SRR6833338	SCFM2	Model	2A, S10
SRR2174566	SCFM	Model	2A, S10
SRR2174567	SCFM	Model	2A, S10
SRR2288354	SCFM	Model	2A, S10
SRR2288355	SCFM	Model	2A, S10
SRR2288356	SCFM	Model	2A, S10
SRR2288357	SCFM	Model	2A, S10
SRR1173936	Mouse tumor	Model	2A, S10
SRR1173937	Mouse tumor	Model	2A, S10
SRR4279873	Mouse pneumonia	Model	2A, S10
SRR4279874	Mouse pneumonia	Model	2A, S10
SRR4279875	Mouse pneumonia	Model	2A, S10
SRR10267776	Airway epithelial cells	Model	2A, S10
SRR10267775	Airway epithelial cells	Model	2A, S10
SRR10267768	Airway epithelial cells	Model	2A, S10
SRR10267767	Airway epithelial cells	Model	2A, S10

Accession*	Description	Type	Used in Fig.
SRR1047563	MOPS succinate	In vitro	1AB, 2A, S10
SRR1047579	MOPS succinate	In vitro	1AB, 2A, S10
ERR3559051	MOPS Glycerol	In vitro	1AB, 2A, S10
ERR3559052	MOPS Glycerol	In vitro	1AB, 2A, S10
ERR3559053	MOPS Glycerol	In vitro	1AB, 2A, S10
ERR3559047	MOPS Acetate	In vitro	1AB, 2A, S10
ERR3559048	MOPS Acetate	In vitro	1AB, 2A, S10
ERR3559049	MOPS Acetate	In vitro	1AB, 2A, S10
ERR2204833	BHI	In vitro	1AB, 2A, S10
ERR2204834	BHI	In vitro	1AB, 2A, S10
SRR8377190	LB	In vitro	1AB, 2A, S10
SRR8377191	LB	In vitro	1AB, 2A, S10
SRR1173924	Biofilm	In vitro	1AB, 2A, S10
SRR1173925	Biofilm	In vitro	1AB, 2A, S10
SRR1173926	Biofilm	In vitro	1AB, 2A, S10
SRR1173927	Biofilm	In vitro	1AB, 2A, S10
SRR2171889	CDM	In vitro	1AB, 2A, S10
SRR2172032	CDM	In vitro	1AB, 2A, S10
SRR7473182	M9 casamino acids	In vitro	1AB, 2A, S10
SRR7473185	M9 casamino acids	In vitro	1AB, 2A, S10
SRR1173956	Stational planktonic	In vitro	1AB, 2A, S10
SRR1173957	Stational planktonic	In vitro	1AB, 2A, S10
SRR1173930	Exponential planktonic	In vitro	1AB, 2A, S10
SRR1173931	Exponential planktonic	In vitro	1AB, 2A, S10
SRR1173920	M9	In vitro	1AB, 2A, S10
SRR1173921	M9	In vitro	1AB, 2A, S10
SRR2153385	Anaerobic	In vitro	2A, S10
SRR2153386	Anaerobic	In vitro	2A, S10
SRR2153387	Anaerobic	In vitro	2A, S10
SRR2153388	Anaerobic	In vitro	2A, S10
SRR1173918	Anaerobic	In vitro	2A, S10
SRR1173919	Anaerobic	In vitro	2A, S10
SRR2007326	anr control	In vitro	2A, S10
SRR2007327	anr control	In vitro	2A, S10
SRR2007328	anr mutant	In vitro	2A, S10
SRR2007329	anr mutant	In vitro	2A, S10
SRR3472878	Calprotectin	In vitro	2A, S10
SRR3472876	Calprotectin	In vitro	2A, S10
SRR3472877	Calprotectin	In vitro	2A, S10
SRR3472875	Calprotectin control	In vitro	2A, S10
SRR3472874	Calprotectin control	In vitro	2A, S10
SRR3472873	Calprotectin control	In vitro	2A, S10
SRR2163805	Carbenicillin	In vitro	2A, S10
SRR2163865	Carbenicillin	In vitro	2A, S10
SRR9587070	Carbon-rich	In vitro	2A, S10
SRR9587071	Carbon-rich	In vitro	2A, S10
SRR9587068	Carbon-rich	In vitro	2A, S10
SRR9587072	Carbon starvation	In vitro	2A, S10
SRR9587073	Carbon starvation	In vitro	2A, S10
SRR9587069	Carbon starvation	In vitro	2A, S10
SRR3468968	c-di-GMP control	In vitro	2A, S10
SRR3468934	c-di-GMP control	In vitro	2A, S10
SRR3468974	c-di-GMP control	In vitro	2A, S10
SRR3468932	c-di-GMP low	In vitro	2A, S10
SRR3468929	c-di-GMP low	In vitro	2A, S10
SRR3468931	c-di-GMP low	In vitro	2A, S10

Supplementary Table 1. (continued)

Accession*	Description	Type	Used in Fig.
SRR2163866	Cefoperazone	In vitro	2A, S10
SRR2164195	Cefoperazone	In vitro	2A, S10
SRR5724640	DNA	In vitro	2A, S10
SRR5724639	DNA	In vitro	2A, S10
SRR2164203	Gentamicin	In vitro	2A, S10
SRR2164205	Gentamicin	In vitro	2A, S10
SRR7473181	Glucose	In vitro	2A, S10
SRR7473184	Glucose	In vitro	2A, S10
SRR10008982	Glycan	In vitro	2A, S10
SRR10008985	Glycan	In vitro	2A, S10
SRR10008993	Glycan	In vitro	2A, S10
SRR10008994	Glycan	In vitro	2A, S10
SRR10008987	Glycan	In vitro	2A, S10
SRR10008988	Glycan	In vitro	2A, S10
SRR10008990	Glycan	In vitro	2A, S10
SRR10008991	Glycan	In vitro	2A, S10
SRR1173942	Heat shock	In vitro	2A, S10
SRR1173941	Heat shock	In vitro	2A, S10
SRR1173938	Heat shock	In vitro	2A, S10
SRR1173939	Heat shock	In vitro	2A, S10
ERR3597223	Hypoxia	In vitro	2A, S10
ERR3597224	Hypoxia	In vitro	2A, S10
ERR3597225	Hypoxia	In vitro	2A, S10
SRR2153381	Hypoxia	In vitro	2A, S10
SRR2153382	Hypoxia	In vitro	2A, S10
SRR2153383	Hypoxia	In vitro	2A, S10
SRR2153384	Hypoxia	In vitro	2A, S10
SRR1173945	Iron starvation	In vitro	2A, S10
SRR1173948	Iron starvation	In vitro	2A, S10
SRR1173949	Iron starvation	In vitro	2A, S10
SRR5724637	L-Asp	In vitro	2A, S10
SRR5724636	L-Asp	In vitro	2A, S10
SRR1173950	Low iron	In vitro	2A, S10
SRR1173951	Low iron	In vitro	2A, S10
SRR1173944	Low iron	In vitro	2A, S10
SRR1173952	Low osmolarity	In vitro	2A, S10
SRR1173953	Low osmolarity	In vitro	2A, S10
SRR1173928	Low phosphorus	In vitro	2A, S10
SRR1173929	Low phosphorus	In vitro	2A, S10
SRR8377186	oligosaccharide	In vitro	2A, S10
SRR8377187	oligosaccharide	In vitro	2A, S10
SRR8377189	oligosaccharide	In vitro	2A, S10
SRR8377188	oligosaccharide	In vitro	2A, S10
SRR4279868	PIA	In vitro	2A, S10
SRR4279869	PIA	In vitro	2A, S10
SRR2164263	Polymyxin B	In vitro	2A, S10
SRR2164280	Polymyxin B	In vitro	2A, S10
SRR11336702	relA-spoT control	In vitro	2A, S10
SRR11336703	relA-spoT control	In vitro	2A, S10
SRR11336705	relA-spoT mutant	In vitro	2A, S10
SRR11336706	relA-spoT mutant	In vitro	2A, S10
SRR8832549	Shear flow	In vitro	2A, S10
SRR8832547	Shear flow	In vitro	2A, S10
SRR8832544	Shear flow	In vitro	2A, S10
SRR8832548	Shear flow	In vitro	2A, S10
SRR8832542	Shear flow	In vitro	2A, S10
SRR1168632	Sigma factor AlgU	In vitro	2A, S10
SRR1168633	Sigma factor AlgU	In vitro	2A, S10
SRR1168639	Sigma factor FecI	In vitro	2A, S10
SRR1168640	Sigma factor FecI	In vitro	2A, S10
SRR1168642	Sigma factor FecI2	In vitro	2A, S10
SRR1168643	Sigma factor FecI2	In vitro	2A, S10
SRR1168649	Sigma factor FliA	In vitro	2A, S10
SRR1168650	Sigma factor FliA	In vitro	2A, S10
SRR1168656	Sigma factor FpvI	In vitro	2A, S10
SRR1168657	Sigma factor FpvI	In vitro	2A, S10
SRR1168662	Sigma factor PvdS	In vitro	2A, S10
SRR1168663	Sigma factor PvdS	In vitro	2A, S10
SRR1173932	Sigma factor RpoD	In vitro	2A, S10
SRR1168667	Sigma factor RpoH	In vitro	2A, S10
SRR1168668	Sigma factor RpoH	In vitro	2A, S10
SRR1168676	Sigma factor RpoN	In vitro	2A, S10
SRR1173962	Sigma factor RpoN	In vitro	2A, S10
SRR1173961	Sigma factor RpoN	In vitro	2A, S10
SRR1173959	Sigma factor RpoS	In vitro	2A, S10
SRR1168683	Sigma factor RpoS	In vitro	2A, S10
SRR1168684	Sigma factor RpoS	In vitro	2A, S10
SRR1173958	Sigma factor RpoS	In vitro	2A, S10
SRR1168693	Sigma factor SigX	In vitro	2A, S10
SRR1168694	Sigma factor SigX	In vitro	2A, S10
SRR2163624	Silver nitrate	In vitro	2A, S10
SRR2163629	Silver nitrate	In vitro	2A, S10
SRR1173922	Static attachment	In vitro	2A, S10
SRR1173923	Static attachment	In vitro	2A, S10
SRR1813215	sutA mutant	In vitro	2A, S10
SRR1813216	sutA mutant	In vitro	2A, S10
SRR1813218	sutA overexpress	In vitro	2A, S10
SRR1813219	sutA overexpress	In vitro	2A, S10
SRR1813212	sutA WT	In vitro	2A, S10
SRR1813213	sutA WT	In vitro	2A, S10

*Additional information regarding each experiment can be found on https://www.ncbi.nlm.nih.gov/sra with the corresponding accession #.

Supplementary Table 2. Candidate SicX-regulated proteins identified in quantitative proteomic analysis. Proteins involved in anaerobic ubiquinone biosynthesis are highlighted in blue. An empirical Bayes moderated *t*-test was used to determine the *P* values.

Seven differentially expressed proteins identified under both static and $-O_2$ conditions				
PA14 locus	PAO1 locus	Protein name	Log_2 fold change (static)	Log_2 fold change ($-O_2$)
PA14_02730	*PA0223*		4.444671	2.14923
PA14_02740	*PA0224*		3.851102	1.60466
PA14_66880	*PA5061*		2.106037	1.44977
PA14_06870	*PA0527*	Dnr	1.415853	0.782226
PA14_00280	*PA0024*	HemF	1.212775	0.7714
PA14_13300	*PA3912*	UbiV	-1.35647	-0.72015
PA14_11140	*PA4078*		-1.85627	-1.61675

Differentially expressed proteins under static conditions: Δ*sicX* vs. WT				
PA14 locus	PAO1 locus	Protein name	Log_2 fold change	Adjusted *P*
PA14_11140	*PA4078*		-1.85627	2.03E-10
PA14_49080	*PA1187*		-1.57572	2.28E-06
PA14_18150	*PA3568*		-1.46312	1.43E-08
PA14_13290	*PA3913*	UbiU	-1.4202	1.03E-09
PA14_67350	*PA5100*	HutU	-1.39602	2.87E-07
PA14_13300	*PA3912*	UbiV	-1.35647	9.43E-11
PA14_05060	*PA0388*		-1.21168	5.76E-07
PA14_49850	*PA1123*		-1.20069	2.57E-08
PA14_39700	N/A		-1.0724	2.8E-08
PA14_36820	*PA2146*		-1.02547	3.86E-05
*PA14_13320**	*PA3911**	UbiT*	-0.82683*	1.05E-06*
PA14_61000	*PA4610*		1.014465	0.000016
PA14_13010	*PA3931*		1.014801	6.33E-06
PA14_01290	*PA0105*	CoxB	1.027092	5.44E-07
PA14_38530	*PA2008*	FahA	1.038382	1.08E-08
PA14_09570	*PA4203*	NmoR	1.039144	3.03E-05
PA14_43600	*PA1618*		1.062549	4.64E-09
PA14_09290	*PA4224*	PchG	1.099311	2.27E-05
PA14_00290	*PA0025*	AroE	1.166368	2.5E-08
PA14_00280	*PA0024*	HemF	1.212775	3.03E-10
PA14_06870	*PA0527*	Dnr	1.415853	1.06E-10
PA14_17930	*PA3584*	GlpD	1.431571	7.57E-09
PA14_09280	*PA4225*	PchF	1.594542	4.64E-09
PA14_00690	*PA0057*		1.624441	5.76E-07
PA14_09220	*PA4230*	PchB	2.001902	5.76E-07
PA14_66880	*PA5061*		2.106037	5.91E-09
PA14_09270	*PA4226*	PchE	2.159572	5.91E-09
PA14_26310	*PA2918*		2.327448	1.54E-08
PA14_09240	*PA4228*	PchD	2.507398	1.02E-07
PA14_09210	*PA4231*	PchA	2.636392	1.08E-08
PA14_02740	*PA0224*		3.851102	1.08E-08
PA14_02730	*PA0223*		4.444671	2.04E-08

Differentially expressed proteins with |fold change| > 2, *P* < 0.01; UbiT is also shown (*)

Differentially expressed proteins under $-O_2$ conditions: Δ*sicX* vs. WT				
PA14 locus	PAO1 locus	Protein name	Log_2 fold change	Adjusted *P*
PA14_11140	*PA4078*		-1.61675	0.050261
PA14_20200	*PA3392*	NosZ	-0.86048	0.009501
PA14_20170	*PA3395*	NosY	-0.77206	0.013393
PA14_13300	*PA3912*	UbiV	-0.72015	0.028161
PA14_20180	*PA3394*	NosF	-0.62216	0.034773
PA14_00280	*PA0024*	HemF	0.7714	0.03572

PA14_06870	*PA0527*	Dnr	0.782226	0.009501
PA14_06890	*PA0529*		0.889094	0.013393
PA14_66880	*PA5061*		1.44977	0.006933
PA14_02740	*PA0224*		1.60466	0.000327
PA14_06900	N/A		1.704533	0.001033
PA14_02730	*PA0223*		2.14923	0.000327

Differentially expressed proteins with |fold change| > 1.5, $P < 0.05$

Supplementary Table 3. Strains, plasmids, and primers used in this study.

Strain name	Description	Source
DH5α	*E. coli* cloning strain	Zymo
SM10 λpir	*E. coli* donor strain	[1]
P. aeruginosa PA14	Wild-type laboratory strain	[2]
P. aeruginosa PAO1	Wild-type laboratory strain	[3]
PA14 P_{sicX}-*lacZ*	*sicX* promoter-*lacZ* transcriptional fusion (mini-CTX based chromosomal integration) in PA14; Tet^R	This study
PA14 $P_{sicX(anr)}$-*lacZ*	*sicX* promoter-*lacZ* (containing mutated Anr binding motif; mini-CTX based chromosomal integration) transcriptional fusion in PA14; Tet^R	This study
anr::MrT7 P_{sicX}-*lacZ*	*sicX* promoter-*lacZ* transcriptional fusion (mini-CTX based chromosomal integration) in *anr*::MrT7 (MAR2xT7 transposon insertion in *anr* in PA14); Tet^R, Gen^R	This study
PA14 Δ*sicX*	Deletion of *sicX* in PA14	This study
PAO1 Δ*sicX*	Deletion of *sicX* in PAO1	This study
B1	A CF clinical isolate	
B1 Δ*sicX*	Deletion of *sicX* in B1	This study
PA14 mini-CTX1	Chromosomal integration of mini-CTX1 in PA14; Tet^R	This study
Δ*sicX* mini-CTX1	Chromosomal integration of mini-CTX1 in Δ*sicX*; Tet^R	This study
Δ*sicX* $sicX^{GCG}$	Δ*sicX* complemented with a mutant *sicX* allele (annotated start codon GTG changed to GCG; mini-CTX1 based chromosomal integration); Tet^R	This study
Δ*sicX* $sicX^{Frameshift}$	Δ*sicX* complemented with a mutant *sicX* allele (frameshift introduced by changing the annotated start codon GTG to GTGT; mini-CTX1 based chromosomal integration); Tet^R	This study
Δ*sicX* $sicX^{Silent}$	Δ*sicX* complemented with a mutant *sicX* allele (silent or synonymous mutation; mini-CTX1 based chromosomal integration); Tet^R	This study
Δ*sicX* $sicX^{Truncate}$	Δ*sicX* complemented with a mutant *sicX* allele (truncated allele containing first 100 bp of the annotated ORF; mini-CTX1 based chromosomal integration); Tet^R	This study
PA14 P_{ubi}-*lacZ* (transl.)	*ubiUVT* promoter-*lacZ* translational fusion (pSW205 plasmid-based expression) in PA14; Cb^R	This study
Δ*sicX* P_{ubi}-*lacZ* (transl.)	*ubiUVT* promoter-*lacZ* translational fusion (pSW205 plasmid-based expression) in Δ*sicX*; Cb^R	This study
Δ*sicX* $sicX^{Truncate}$ P_{ubi}-*lacZ*	*ubiUVT* promoter-*lacZ* translational fusion (pSW205 plasmid-based expression) in Δ*sicX* $sicX^{Truncate}$; Cb^R, Tet^R	This study
Δ*sicX* mini-CTX1 P_{ubi}-*lacZ*	*ubiUVT* promoter-*lacZ* translational fusion (pSW205 plasmid-based expression) in Δ*sicX* with empty mini-CTX1 vector; Cb^R, Tet^R	This study
Δ*sicX* $sicX^{A2T}$ P_{ubi}-*lacZ*	*ubiUVT* promoter-*lacZ* translational fusion (pSW205 plasmid-based expression) in Δ*sicX*, complemented with a *sicX* point mutant (A2T; mini-CTX1 based chromosomal integration); Cb^R, Tet^R	This study
Δ*sicX* $sicX^{A5T}$ P_{ubi}-*lacZ*	*ubiUVT* promoter-*lacZ* translational fusion (pSW205 plasmid-based expression) in Δ*sicX*, complemented with a *sicX* point mutant (A5T; mini-CTX1 based chromosomal integration); Cb^R, Tet^R	This study
Δ*sicX* $sicX^{G8C}$ P_{ubi}-*lacZ*	*ubiUVT* promoter-*lacZ* translational fusion (pSW205 plasmid-based expression) in Δ*sicX*, complemented with a *sicX* point mutant (G8C; mini-CTX1 based chromosomal integration); Cb^R, Tet^R	This study
Δ*sicX* $sicX^{T11A}$ P_{ubi}-*lacZ*	*ubiUVT* promoter-*lacZ* translational fusion (pSW205 plasmid-based expression) in Δ*sicX*, complemented with a *sicX* point mutant (T11A; mini-CTX1 based chromosomal integration); Cb^R, Tet^R	This study
Δ*sicX* $sicX^{A14T}$ P_{ubi}-*lacZ*	*ubiUVT* promoter-*lacZ* translational fusion (pSW205 plasmid-based expression) in Δ*sicX*, complemented with a *sicX* point mutant (A14T; mini-CTX1 based chromosomal integration); Cb^R, Tet^R	This study
Δ*sicX* $sicX^{A15T}$ P_{ubi}-*lacZ*	*ubiUVT* promoter-*lacZ* translational fusion (pSW205 plasmid-based expression) in Δ*sicX*, complemented with a *sicX* point mutant (A15T; mini-CTX1 based chromosomal integration); Cb^R, Tet^R	This study
Δ*sicX* $sicX^{C16G}$ P_{ubi}-*lacZ*	*ubiUVT* promoter-*lacZ* translational fusion (pSW205 plasmid-based expression) in Δ*sicX*, complemented with a *sicX* point mutant (C16G; mini-CTX1 based chromosomal integration); Cb^R, Tet^R	This study

Δ*sicX* *sicX*^{G17C} P$_{ubi}$-*lacZ*	*ubiUVT* promoter-*lacZ* translational fusion (pSW205 plasmid-based expression) in Δ*sicX*, complemented with a *sicX* point mutant (G17C; mini-CTX1 based chromosomal integration); Cb^{R}, Tet^{R}	This study
Δ*sicX* *sicX*^{T18A} P$_{ubi}$-*lacZ*	*ubiUVT* promoter-*lacZ* translational fusion (pSW205 plasmid-based expression) in Δ*sicX*, complemented with a *sicX* point mutant (T18A; mini-CTX1 based chromosomal integration); Cb^{R}, Tet^{R}	This study
Δ*sicX* *sicX*^{G19C} P$_{ubi}$-*lacZ*	*ubiUVT* promoter-*lacZ* translational fusion (pSW205 plasmid-based expression) in Δ*sicX*, complemented with a *sicX* point mutant (G19C; mini-CTX1 based chromosomal integration); Cb^{R}, Tet^{R}	This study
Δ*sicX* *sicX*^{C20G} P$_{ubi}$-*lacZ*	*ubiUVT* promoter-*lacZ* translational fusion (pSW205 plasmid-based expression) in Δ*sicX*, complemented with a *sicX* point mutant (C20G; mini-CTX1 based chromosomal integration); Cb^{R}, Tet^{R}	This study
Δ*sicX* *sicX*^{G21C} P$_{ubi}$-*lacZ*	*ubiUVT* promoter-*lacZ* translational fusion (pSW205 plasmid-based expression) in Δ*sicX*, complemented with a *sicX* point mutant (G21C; mini-CTX1 based chromosomal integration); Cb^{R}, Tet^{R}	This study
Δ*sicX* *sicX*^{G22C} P$_{ubi}$-*lacZ*	*ubiUVT* promoter-*lacZ* translational fusion (pSW205 plasmid-based expression) in Δ*sicX*, complemented with a *sicX* point mutant (G22C; mini-CTX1 based chromosomal integration); Cb^{R}, Tet^{R}	This study
Δ*sicX* *sicX*^{A23T} P$_{ubi}$-*lacZ*	*ubiUVT* promoter-*lacZ* translational fusion (pSW205 plasmid-based expression) in Δ*sicX*, complemented with a *sicX* point mutant (A23T; mini-CTX1 based chromosomal integration); Cb^{R}, Tet^{R}	This study
Δ*sicX* *sicX*^{G24C} P$_{ubi}$-*lacZ*	*ubiUVT* promoter-*lacZ* translational fusion (pSW205 plasmid-based expression) in Δ*sicX*, complemented with a *sicX* point mutant (G24C; mini-CTX1 based chromosomal integration); Cb^{R}, Tet^{R}	This study
Δ*sicX* *sicX*^{A25T} P$_{ubi}$-*lacZ*	*ubiUVT* promoter-*lacZ* translational fusion (pSW205 plasmid-based expression) in Δ*sicX*, complemented with a *sicX* point mutant (A25T; mini-CTX1 based chromosomal integration); Cb^{R}, Tet^{R}	This study
Δ*sicX* *sicX*^{G26C} P$_{ubi}$-*lacZ*	*ubiUVT* promoter-*lacZ* translational fusion (pSW205 plasmid-based expression) in Δ*sicX*, complemented with a *sicX* point mutant (G26C; mini-CTX1 based chromosomal integration); Cb^{R}, Tet^{R}	This study
Δ*sicX* *sicX*^{T27A} P$_{ubi}$-*lacZ*	*ubiUVT* promoter-*lacZ* translational fusion (pSW205 plasmid-based expression) in Δ*sicX*, complemented with a *sicX* point mutant (T27A; mini-CTX1 based chromosomal integration); Cb^{R}, Tet^{R}	This study
Δ*sicX* *sicX*^{Truncate} P$_{ubi}^{A1T}$-*lacZ*	*ubiUVT* promoter-*lacZ* translational fusion (A1T; pSW205 plasmid-based expression) in Δ*sicX* *sicX*^{Truncate}; Cb^{R}, Tet^{R}	This study
Δ*sicX* *sicX*^{Truncate} P$_{ubi}^{G2C}$-*lacZ*	*ubiUVT* promoter-*lacZ* translational fusion (G2C; pSW205 plasmid-based expression) in Δ*sicX* *sicX*^{Truncate}; Cb^{R}, Tet^{R}	This study
Δ*sicX* *sicX*^{Truncate} P$_{ubi}^{C3G}$-*lacZ*	*ubiUVT* promoter-*lacZ* translational fusion (C3G; pSW205 plasmid-based expression) in Δ*sicX* *sicX*^{Truncate}; Cb^{R}, Tet^{R}	This study
Δ*sicX* *sicX*^{Truncate} P$_{ubi}^{G4C}$-*lacZ*	*ubiUVT* promoter-*lacZ* translational fusion (G4C; pSW205 plasmid-based expression) in Δ*sicX* *sicX*^{Truncate}; Cb^{R}, Tet^{R}	This study
Δ*sicX* *sicX*^{Truncate} P$_{ubi}^{C5G}$-*lacZ*	*ubiUVT* promoter-*lacZ* translational fusion (C5G; pSW205 plasmid-based expression) in Δ*sicX* *sicX*^{Truncate}; Cb^{R}, Tet^{R}	This study
Δ*sicX* *sicX*^{Truncate} P$_{ubi}^{G6C}$-*lacZ*	*ubiUVT* promoter-*lacZ* translational fusion (G6C; pSW205 plasmid-based expression) in Δ*sicX* *sicX*^{Truncate}; Cb^{R}, Tet^{R}	This study
Δ*sicX* *sicX*^{Truncate} P$_{ubi}^{T7A}$-*lacZ*	*ubiUVT* promoter-*lacZ* translational fusion (T7A; pSW205 plasmid-based expression) in Δ*sicX* *sicX*^{Truncate}; Cb^{R}, Tet^{R}	This study
Δ*sicX* *sicX*^{Truncate} P$_{ubi}^{T8A}$-*lacZ*	*ubiUVT* promoter-*lacZ* translational fusion (T8A; pSW205 plasmid-based expression) in Δ*sicX* *sicX*^{Truncate}; Cb^{R}, Tet^{R}	This study
Δ*sicX* *sicX*^{Truncate} P$_{ubi}^{T9A}$-*lacZ*	*ubiUVT* promoter-*lacZ* translational fusion (T9A; pSW205 plasmid-based expression) in Δ*sicX* *sicX*^{Truncate}; Cb^{R}, Tet^{R}	This study
Δ*sicX* *sicX*^{Truncate} P$_{ubi}^{T10A}$-*lacZ*	*ubiUVT* promoter-*lacZ* translational fusion (T10A; pSW205 plasmid-based expression) in Δ*sicX* *sicX*^{Truncate}; Cb^{R}, Tet^{R}	This study
Δ*sicX* *sicX*^{Truncate} P$_{ubi}^{T11A}$-*lacZ*	*ubiUVT* promoter-*lacZ* translational fusion (T11A; pSW205 plasmid-based expression) in Δ*sicX* *sicX*^{Truncate}; Cb^{R}, Tet^{R}	This study
Δ*sicX* *sicX*^{Truncate} P$_{ubi}^{C12G}$-*lacZ*	*ubiUVT* promoter-*lacZ* translational fusion (C12G; pSW205 plasmid-based expression) in Δ*sicX* *sicX*^{Truncate}; Cb^{R}, Tet^{R}	This study
Δ*sicX* *sicX*^{Truncate} P$_{ubi}^{G13C}$-*lacZ*	*ubiUVT* promoter-*lacZ* translational fusion (G13C; pSW205 plasmid-based expression) in Δ*sicX* *sicX*^{Truncate}; Cb^{R}, Tet^{R}	This study
Δ*sicX* *sicX*^{Truncate} P$_{ubi}^{C14G}$-*lacZ*	*ubiUVT* promoter-*lacZ* translational fusion (C14G; pSW205 plasmid-based expression) in Δ*sicX* *sicX*^{Truncate}; Cb^{R}, Tet^{R}	This study

Δ*sicX* *sicX*^{Truncate} P$_{ubi}^{C15G}$-*lacZ*	*ubiUVT* promoter-*lacZ* translational fusion (C15G; pSW205 plasmid-based expression) in Δ*sicX* *sicX*^{Truncate}; Cb^{R}, Tet^{R}	This study
Δ*sicX* *sicX*^{Truncate} P$_{ubi}^{C23G}$-*lacZ*	*ubiUVT* promoter-*lacZ* translational fusion (C23G; pSW205 plasmid-based expression) in Δ*sicX* *sicX*^{Truncate}; Cb^{R}, Tet^{R}	This study
Δ*sicX* *sicX*^{Truncate} P$_{ubi}^{A24T}$-*lacZ*	*ubiUVT* promoter-*lacZ* translational fusion (A24T; pSW205 plasmid-based expression) in Δ*sicX* *sicX*^{Truncate}; Cb^{R}, Tet^{R}	This study
Δ*sicX* *sicX*^{Truncate} P$_{ubi}^{A25T}$-*lacZ*	*ubiUVT* promoter-*lacZ* translational fusion (A25T; pSW205 plasmid-based expression) in Δ*sicX* *sicX*^{Truncate}; Cb^{R}, Tet^{R}	This study
Δ*sicX* *sicX*^{Truncate} P$_{ubi}^{C26G}$-*lacZ*	*ubiUVT* promoter-*lacZ* translational fusion (C26G; pSW205 plasmid-based expression) in Δ*sicX* *sicX*^{Truncate}; Cb^{R}, Tet^{R}	This study
Δ*sicX* *sicX*^{C16G} P$_{ubi}^{G6C}$-*lacZ*	*ubiUVT* promoter-*lacZ* translational fusion (G6C; pSW205 plasmid-based expression) in Δ*sicX*, complemented with a *sicX* point mutant (C16G; mini-CTX1 based chromosomal integration); Cb^{R}, Tet^{R}	This study
PA14 Δ*hfq* P$_{ubi}$-*lacZ*	*ubiUVT* promoter-*lacZ* translational fusion (pSW205 plasmid-based expression) in Δ*hfq*; Cb^{R}	This study
Δ*sicX* Δ*hfq* P$_{ubi}$-*lacZ*	*ubiUVT* promoter-*lacZ* translational fusion (pSW205 plasmid-based expression) in Δ*sicX* Δ*hfq*; Cb^{R}	This study
PA14 Δ*ubiUVT*	Deletion of *ubiUVT* in PA14	This study
PA14 P$_{ubi}$-*lacZ* (transcr.)	*ubiUVT* promoter-*lacZ* transcriptional fusion (mini-CTX based chromosomal integration) in WT; Tet^{R}	This study
Δ*sicX* P$_{ubi}$-*lacZ* (transcr.)	*ubiUVT* promoter-*lacZ* transcriptional fusion (mini-CTX based chromosomal integration) in Δ*sicX*; Tet^{R}	This study
Δ*anr* P$_{ubi}$-*lacZ* (transcr.)	*ubiUVT* promoter-*lacZ* transcriptional fusion (mini-CTX based chromosomal integration) in Δ*anr*; Tet^{R}	This study
PA14 P$_{ubi}^{c}$-*lacZ*	Constitutive *ubiUVT* promoter-*lacZ* translational fusion (pSW205 plasmid-based expression) in WT; Cb^{R}	This study
Δ*sicX* P$_{ubi}^{c}$-*lacZ*	Constitutive *ubiUVT* promoter-*lacZ* translational fusion (pSW205 plasmid-based expression) in Δ*sicX*; Cb^{R}	This study
Δ*sicX*-*ubi*^{c}	Constitutive *ubiUVT* promoter in Δ*sicX*	This study
gacS::MrT7 P$_{sicX}$-*lacZ*	*sicX* promoter-*lacZ* transcriptional fusion (mini-CTX based chromosomal integration) in *gacS*::MrT7 (MAR2xT7 transposon insertion in *gacS* in PA14); Tet^{R}, Gen^{R}	This study
gacA::MrT7 P$_{sicX}$-*lacZ*	*sicX* promoter-*lacZ* transcriptional fusion (mini-CTX based chromosomal integration) in *gacA*::MrT7 (MAR2xT7 transposon insertion in *gacA* in PA14); Tet^{R}, Gen^{R}	This study

Plasmid name	Description	Source
mini-CTX-*lacZ*	Parental vector for creating transcriptional *lacZ* fusion; Tet^{R}	[4]
pPC100	mini-CTX-P$_{sicX}$-*lacZ*; *sicX* promoter transcriptionally fused with *lacZ*; Tet^{R}	This study
pPC101	mini-CTX-P$_{sicX(anr)}$-*lacZ*; *sicX* promoter (containing mutated Anr binding motif) transcriptionally fused with *lacZ*; Tet^{R}	This study
pPC102	mini-CTX-P$_{ubi}$-*lacZ*; *ubiUVT* promoter transcriptionally fused with *lacZ*; Tet^{R}	This study
mini-CTX1	Parental integration plasmid; Tet^{R}	[5]
pPC103	mini-CTX1-*sicX*^{GCG}; *sicX* harboring a start codon mutation (annotated start codon GTG changed to GCG); Tet^{R}	This study
pPC104	mini-CTX1-*sicX*^{Frameshift}; *sicX* harboring a frameshift mutation (frameshift introduced by changing the annotated start codon GTG to GTGT); Tet^{R}	This study
pPC105	mini-CTX1-*sicX*^{Silent}; *sicX* harboring silent or synonymous mutations; Tet^{R}	This study
pPC106	mini-CTX1-*sicX*^{Truncate}; truncated *sicX* allele containing first 100 bp of the annotated ORF; Tet^{R}	This study
pPC107	mini-CTX1-*sicX*^{A2T}; point mutation of *sicX* allele; Tet^{R}	This study
pPC108	mini-CTX1-*sicX*^{A5T}; point mutation of *sicX* allele; Tet^{R}	This study
pPC109	mini-CTX1-*sicX*^{G8C}; point mutation of *sicX* allele; Tet^{R}	This study
pPC110	mini-CTX1-*sicX*^{T11A}; point mutation of *sicX* allele; Tet^{R}	This study
pPC111	mini-CTX1-*sicX*^{A14T}; point mutation of *sicX* allele; Tet^{R}	This study
pPC112	mini-CTX1-*sicX*^{A15T}; point mutation of *sicX* allele; Tet^{R}	This study

pPC113	mini-CTX1-*sicX*^{C16G}; point mutation of *sicX* allele; Tet^{R}	This study
pPC114	mini-CTX1-*sicX*^{G17C}; point mutation of *sicX* allele; Tet^{R}	This study
pPC115	mini-CTX1-*sicX*^{T18A}; point mutation of *sicX* allele; Tet^{R}	This study
pPC116	mini-CTX1-*sicX*^{G19C}; point mutation of *sicX* allele; Tet^{R}	This study
pPC117	mini-CTX1-*sicX*^{C20G}; point mutation of *sicX* allele; Tet^{R}	This study
pPC118	mini-CTX1-*sicX*^{G21C}; point mutation of *sicX* allele; Tet^{R}	This study
pPC119	mini-CTX1-*sicX*^{G22C}; point mutation of *sicX* allele; Tet^{R}	This study
pPC120	mini-CTX1-*sicX*^{A23T}; point mutation of *sicX* allele; Tet^{R}	This study
pPC121	mini-CTX1-*sicX*^{G24C}; point mutation of *sicX* allele; Tet^{R}	This study
pPC122	mini-CTX1-*sicX*^{A25T}; point mutation of *sicX* allele; Tet^{R}	This study
pPC123	mini-CTX1-*sicX*^{G26C}; point mutation of *sicX* allele; Tet^{R}	This study
pPC124	mini-CTX1-*sicX*^{T27A}; point mutation of *sicX* allele; Tet^{R}	This study
pSW205	Parental vector for creating translational *lacZ* fusion; Cb^{R}	[6]
pPC125	pSW205-P$_{ubi}$-*lacZ*; *ubiUVT* promoter translationally fused with *lacZ* fusion; Cb^{R}	This study
pPC126	pSW205-P$_{ubi}^{c}$-*lacZ*; constitutive *ubiUVT* promoter translationally fused with *lacZ* fusion; Cb^{R}	This study
pPC127	pSW205-P$_{ubi}^{A1T}$-*lacZ*; *ubiUVT* promoter (containing a point mutation) translationally fused with *lacZ* fusion; Cb^{R}	This study
pPC128	pSW205-P$_{ubi}^{G2C}$-*lacZ*; *ubiUVT* promoter (containing a point mutation) translationally fused with *lacZ* fusion; Cb^{R}	This study
pPC129	pSW205-P$_{ubi}^{C3G}$-*lacZ*; *ubiUVT* promoter (containing a point mutation) translationally fused with *lacZ* fusion; Cb^{R}	This study
pPC130	pSW205-P$_{ubi}^{G4C}$-*lacZ*; *ubiUVT* promoter (containing a point mutation) translationally fused with *lacZ* fusion; Cb^{R}	This study
pPC131	pSW205-P$_{ubi}^{C5G}$-*lacZ*; *ubiUVT* promoter (containing a point mutation) translationally fused with *lacZ* fusion; Cb^{R}	This study
pPC132	pSW205-P$_{ubi}^{G6C}$-*lacZ*; *ubiUVT* promoter (containing a point mutation) translationally fused with *lacZ* fusion; Cb^{R}	This study
pPC133	pSW205-P$_{ubi}^{T7A}$-*lacZ*; *ubiUVT* promoter (containing a point mutation) translationally fused with *lacZ* fusion; Cb^{R}	This study
pPC134	pSW205-P$_{ubi}^{T8A}$-*lacZ*; *ubiUVT* promoter (containing a point mutation) translationally fused with *lacZ* fusion; Cb^{R}	This study
pPC135	pSW205-P$_{ubi}^{T9A}$-*lacZ*; *ubiUVT* promoter (containing a point mutation) translationally fused with *lacZ* fusion; Cb^{R}	This study
pPC136	pSW205-P$_{ubi}^{T10A}$-*lacZ*; *ubiUVT* promoter (containing a point mutation) translationally fused with *lacZ* fusion; Cb^{R}	This study
pPC137	pSW205-P$_{ubi}^{T11A}$-*lacZ*; *ubiUVT* promoter (containing a point mutation) translationally fused with *lacZ* fusion; Cb^{R}	This study
pPC138	pSW205-P$_{ubi}^{C12G}$-*lacZ*; *ubiUVT* promoter (containing a point mutation) translationally fused with *lacZ* fusion; Cb^{R}	This study
pPC139	pSW205-P$_{ubi}^{G13C}$-*lacZ*; *ubiUVT* promoter (containing a point mutation) translationally fused with *lacZ* fusion; Cb^{R}	This study
pPC140	pSW205-P$_{ubi}^{C14G}$-*lacZ*; *ubiUVT* promoter (containing a point mutation) translationally fused with *lacZ* fusion; Cb^{R}	This study
pPC141	pSW205-P$_{ubi}^{C15G}$-*lacZ*; *ubiUVT* promoter (containing a point mutation) translationally fused with *lacZ* fusion; Cb^{R}	This study
pPC142	pSW205-P$_{ubi}^{C23G}$-*lacZ*; *ubiUVT* promoter (containing a point mutation) translationally fused with *lacZ* fusion; Cb^{R}	This study
pPC143	pSW205-P$_{ubi}^{A24T}$-*lacZ*; *ubiUVT* promoter (containing a point mutation) translationally fused with *lacZ* fusion; Cb^{R}	This study
pPC144	pSW205-P$_{ubi}^{A25T}$-*lacZ*; *ubiUVT* promoter (containing a point mutation) translationally fused with *lacZ* fusion; Cb^{R}	This study
pPC145	pSW205-P$_{ubi}^{C26G}$-*lacZ*; *ubiUVT* promoter (containing a point mutation) translationally fused with *lacZ* fusion; Cb^{R}	This study
pEXG2	Parental plasmid used markerless DNA mutagenesis; *sacB*, Gen^{R}	[7]
pPC146	pEXG2-Δ*sicX*; *sicX* deletion cassette; *sacB*, Gen^{R}	This study
pPC147	pEXG2-Δ*ubiUVT*; *ubiUVT* deletion cassette; *sacB*, Gen^{R}	This study
pPC148	pEXG2-*ubi*^{C}; constitutive *ubiUVT* promoter cassette; *sacB*, Gen^{R}	This study
pPC149	pEXG2-Δ*hfq*; *hfq* deletion cassette; *sacB*, Gen^{R}	This study

pPC150	pEXG2-Δ*anr*; *anr* deletion cassette; *sacB*, Gen^{R}	This study

Primer name	Sequence (from 5' to 3')
mini-CTX-*sicX* promoter-F	ACGACTCACTATAGGGCGAATTGGGTACCAGCGCGGTGATCGCCTTC
mini-CTX-*sicX* promoter-R	AGGATCCCCCGGGCTGCAGGAATTCCTCCGCACGTTCCACTCCG
sicX promoter *anr*-R	GCCAACGCCGATTCCCGC
sicX promoter *anr*-F	GCAAGGGCGGGAATCGGCGTTGGCCGTGCCAGATCAGAGGTCCGGGGCCGGTTTC
GTG to GCG-R	CCGCACGTTCCGCTCCGTCCTTGGAAACAACTTTACTG
GTG to GCG-F	TTCCAAGGACGGAGCGGAACGTGCGGAGAGTGGG
frameshift-R	CTCCGCACGTTCACACTCCGTCCTTGGAAACAACTTTACTGC
frameshift-F	AAGGACGGAGTGTGAACGTGCGGAGAGTGGGAC
mini-CTX-*sicX*-R	GGATCCCCCGGGCTGCAGGAATTCTCAGCGACGTTGCCGCG
silent mutation-R	GCTTTCGGCTCGCTCCACTCCGTCCTTGGAAACAACTTTACTG
silent mutation-F	CGGAGTGGAGCGAGCCGAAAGCGGACGCCGGTCTACTCT
mini-CTX-*sicX*^{Truncate}-R	GGATCCCCCGGGCTGCAGGAATTCTGACAGGCCCCTTGTCCAAG
mini-CTX-*ubiUVT* promoter-F	ACGACTCACTATAGGGCGAATTGGGTACCGGCCTTCTTGATCCCGGTCAAG
mini-CTX-*ubiUVT* promoter-R	AGGATCCCCCGGGCTGCAGGAATTCGCGCCCTGTCGCAGGGC
pSW205-*ubiUVT* promoter-F	GACGACGAATTCGGCCTTCTTGATCCCGGTCAAG
pSW205-*ubiUVT* promoter-R	GACGACGGATCCGCGCCCTGTCGCAGGGC
pSW205-*ubi*^{c}-F	GTTTTTCGCCAGAGGAGTCCTTTTATGCAACTGGTCTGTCCGG
pSW205-*ubi*^{c}-R	GCATAAAAGGACTCCTCTGGCGAAAAACGC
sicX-upstream-F	CTAGAGGATCCCCGGGCTCGAGCTCGGCAACTGGAGGAACAGC
sicX-upstream-R	CGGGGAACCGGCTCCGTCCTTGGAAACAACTTTACTG
sicX-downstrean-F	GTTTCCAAGGACGGA GCCGGTTCCCCGACGCTCTGGC
sicX-downstrean-R	ACCCGTGGAAATTAATTAAGGTACCATGAGAATCGTCAGCCGCGAC
ubi^{C}-upstream-F	CTAGAGGATCCCCGGGCTCGAGCTCGGCGGCGCGGATCTTCAC
ubi^{C}-upstream-R	CGGACAGACCAGTTGCATAAAAGGACTCCTCTGGCGAAAAACGCGC
ubi^{C}-downstrean-F	CGTTTTTCGCCAGAGGAGTCCTTTTATGCAACTGGTCTGTCCGGC
ubi^{C}-downstrean-R	ACCCGTGGAAATTAATTAAGGTACCTCACTGCCAGGAACGGTGATAGG
ubiU-upstream-F	CTAGAGGATCCCCGGGCTCGAGCTCCGCAGGAACTCTGCGTGGTC
ubiU-upstream-R	TTTCCACCGACCGGGGTTGCTCCTCTGGCG
ubiT-downstream-F	GAGGAGCAACCCCGGTCGGTGGAAAGGGGCGTATA
ubiT-downstream-R	ACCCGTGGAAATTAATTAAGGTACCGTGGTCTCGTGGTCGTCCC
hfq-upstream-F	CTAGAGGATCCCCGGGCTCGAGCTCCTGCGAGCTGATCAGCGT
hfq-upstream-R	GGACTCCCGTCAGTGCCGCACTCCTTTAAGGA
hfq-downstream-F	GGAGTGCGGCACTGACGGGAGTCCGCTTTGTT
hfq-downstream-R	ACCCGTGGAAATTAATTAAGGTACCGTAGCAGGTCGACCTTGTTATAGACC
anr-upstream-F	CTAGAGGATCCCCGGGCTCGAGCTCCTGCCACTTTGAACTGGCC
anr-upstream-R	GCCTTCCAGCTGCTTGATGGTTTCGGCCATTGA
anr-downstream-F	GAAACCATCAAGCAGCTGGAAGGCTGAAGC
anr-downstream-R	ACCCGTGGAAATTAATTAAGGTACCGTCATGAAGGGTTGGCCG
sicX-1 (Northern blot Fig. 2C)	ACTCTCCGCACGTTCCACTCCG
sicX-2 (Northern blot Extended Data Fig. 2F)	CCTGACAAGTCAGGTCGCCCTA
5S rRNA (Northern blot Fig. 2C and Extended Data Fig. 2F)	CGTTTCACTTCTGAGTTCGGGAAGG

All point mutation primers were designed using https://nebasechanger.neb.com. Sequence details are available upon request.

References:

1 Simon, R., Priefer, U. & Pühler, A. A broad host range mobilization system for in vivo genetic engineering: transposon mutagenesis in gram negative bacteria. *Bio/technology* **1**, 784-791 (1983).

2 Rahme, L. G. *et al.* Common virulence factors for bacterial pathogenicity in plants and animals. *Science* **268**, 1899-1902 (1995).

3 Stover, C. K. *et al.* Complete genome sequence of *Pseudomonas aeruginosa* PAO1, an opportunistic pathogen. *Nature* **406**, 959-964 (2000).

4 Becher, A. & Schweizer, H. P. Integration-proficient *Pseudomonas aeruginosa* vectors for isolation of single-copy chromosomal lacZ and lux gene fusions. *Biotechniques* **29**, 948-952 (2000).

5 Hoang, T. T., Kutchma, A. J., Becher, A. & Schweizer, H. P. Integration-proficient plasmids for *Pseudomonas aeruginosa*: site-specific integration and use for engineering of reporter and expression strains. *Plasmid* **43**, 59-72 (2000).

6 West, S., Kaye, S., Hamood, A. & Iglewski, B. Characterization of *Pseudomonas aeruginosa* mutants that are deficient in exotoxin A synthesis and are altered in expression of *regA*, a positive regulator of exotoxin A. *Infection and immunity* **62**, 897-903 (1994).

7 Rietsch, A., Vallet-Gely, I., Dove, S. L. & Mekalanos, J. J. ExsE, a secreted regulator of type III secretion genes in *Pseudomonas aeruginosa*. *Proceedings of the National Academy of Sciences* **102**, 8006-8011 (2005).
