# Supplementary material for: A Pseudomonas aeruginosa small RNA regulates chronic and acute infection
Source: Nature. 2023 May 24;618(7964):358–64. doi: 10.1038/s41586-023-06111-7 (PMC10247376; doi:10.1038/s41586-023-06111-7)
Supplement: Supplementary file 2 — Reporting Summary [file 41586_2023_6111_MOESM2_ESM.pdf]

Corresponding author(s): Marvin Whiteley

Last updated by author(s): Mar 30, 2023

Reporting Summary

Nature Portfolio wishes to improve the reproducibility of the work that we publish. This form provides structure for consistency and transparency in reporting. For further information on Nature Portfolio policies, see our Editorial Policies and the Editorial Policy Checklist.

Statistics

For all statistical analyses, confirm that the following items are present in the figure legend, table legend, main text, or Methods section.

n/a	Confirmed	
☐	☒	The exact sample size (*n*) for each experimental group/condition, given as a discrete number and unit of measurement
☐	☒	A statement on whether measurements were taken from distinct samples or whether the same sample was measured repeatedly
☐	☒	The statistical test(s) used AND whether they are one- or two-sided<br>*Only common tests should be described solely by name; describe more complex techniques in the Methods section.*
☒	☐	A description of all covariates tested
☐	☒	A description of any assumptions or corrections, such as tests of normality and adjustment for multiple comparisons
☐	☒	A full description of the statistical parameters including central tendency (e.g. means) or other basic estimates (e.g. regression coefficient) AND variation (e.g. standard deviation) or associated estimates of uncertainty (e.g. confidence intervals)
☐	☒	For null hypothesis testing, the test statistic (e.g. *F*, *t*, *r*) with confidence intervals, effect sizes, degrees of freedom and *P* value noted<br>*Give P values as exact values whenever suitable.*
☒	☐	For Bayesian analysis, information on the choice of priors and Markov chain Monte Carlo settings
☒	☐	For hierarchical and complex designs, identification of the appropriate level for tests and full reporting of outcomes
☒	☐	Estimates of effect sizes (e.g. Cohen's *d*, Pearson's *r*), indicating how they were calculated

Our web collection on statistics for biologists contains articles on many of the points above.

Software and code

Policy information about availability of computer code

Data collection	No software was used for data collection.
Data analysis	As described in Methods, RNA-seq analyses utilized FastQC v0.11.7, Cutadapt 3.0, Bowtie2 v2.4.2, featureCounts Subread v2.0.1, DESeq2, and samtools v1.13; proteomics analyses utilized Proteome Discoverer v2.1 (Thermo Fisher Scientific), Mascot (Matrix Science) v2.6, R package MSnbase v2.13, and Limma package v3.44.3.

For manuscripts utilizing custom algorithms or software that are central to the research but not yet described in published literature, software must be made available to editors and reviewers. We strongly encourage code deposition in a community repository (e.g. GitHub). See the Nature Portfolio guidelines for submitting code & software for further information.

Data

Policy information about availability of data

All manuscripts must include a data availability statement. This statement should provide the following information, where applicable:

- Accession codes, unique identifiers, or web links for publicly available datasets
- A description of any restrictions on data availability
- For clinical datasets or third party data, please ensure that the statement adheres to our policy

The proteomics datasets generated in this study have been deposited in the ProteomeXchange Consortium (http://proteomecentral.proteomexchange.org/cgi/GetDataset), and the accession numbers are PXD032985 and PXD032986. Raw reads of our RNA-seq studies have been deposited in the Sequence Read Archive

(SRA; https://www.ncbi.nlm.nih.gov/sra) under BioProject PRJNA934328 and PRJNA904394. The 202 publicly available P. aeruginosa transcriptomes analyzed in this study are summarized in Supplementary Table 1, which can be obtained from SRA (https://www.ncbi.nlm.nih.gov/sra) with the corresponding accession number. The P. aeruginosa PA14 reference genome (BioSample Accession: SAMN02603591) is available for download from https://pseudomonas.com.

Human research participants

Policy information about studies involving human research participants and Sex and Gender in Research.

Reporting on sex and gender	No human research participants involved in this study.
Population characteristics	No human research participants involved in this study.
Recruitment	No human research participants involved in this study.
Ethics oversight	No human research participants involved in this study.

Note that full information on the approval of the study protocol must also be provided in the manuscript.

Field-specific reporting

Please select the one below that is the best fit for your research. If you are not sure, read the appropriate sections before making your selection.

☒ Life sciences ☐ Behavioural & social sciences ☐ Ecological, evolutionary & environmental sciences

For a reference copy of the document with all sections, see nature.com/documents/nr-reporting-summary-flat.pdf

Life sciences study design

All studies must disclose on these points even when the disclosure is negative.

Sample size	Exact sample size (n) are provided in the figures and/or figure legends. For all in vitro experiments, $n \geq 4$ and no sample size calculation was conducted as this is an established practice in the field. For animal studies, experiments were conducted at least twice independently with minimal animal numbers in compliance with IACUC guidelines, and the size was estimated based on power analysis of previous/preliminary data.
Data exclusions	No data were excluded when experiments were conducted successfully (i.e. experimental controls worked).
Replication	Experimental findings are reproducible, confirmed by repeating the experiments independently with proper controls. The replication information for each experiment is described in the figure legends.
Randomization	Animals used in this studies were randomly assigned to infections with different bacterial strains. For in vitro experiments, randomization is not relevant as they involved bacterial cells, and for example, equivalent numbers of cells were used for different treatments/conditions.
Blinding	Blinding was not performed because it is not a standard practice for experiments conducted in this study, and all data were generated objectively with replication that showed similar observations.

Reporting for specific materials, systems and methods

We require information from authors about some types of materials, experimental systems and methods used in many studies. Here, indicate whether each material, system or method listed is relevant to your study. If you are not sure if a list item applies to your research, read the appropriate section before selecting a response.

Materials & experimental systems

n/a	Involved in the study	
☒	☐	Antibodies
☒	☐	Eukaryotic cell lines
☒	☐	Palaeontology and archaeology
☐	☒	Animals and other organisms
☒	☐	Clinical data
☒	☐	Dual use research of concern

Methods

n/a	Involved in the study	
☒	☐	ChIP-seq
☒	☐	Flow cytometry
☒	☐	MRI-based neuroimaging

Animals and other research organisms

Policy information about studies involving animals; ARRIVE guidelines recommended for reporting animal research, and Sex and Gender in Research

Laboratory animals	BALB/c mice (female, 6-8 weeks) were used for the mouse pneumonia model. Swiss Webster mice (female, 8-10 weeks) were used for all other animal studies. The mice were housed in facilities with a temperature range of 18–22°C, 40-50% humidity, and a 12-hour light-dark cycle.
Wild animals	No wild animals used in this study.
Reporting on sex	Female animals were used in this study.
Field-collected samples	No field-collected samples used in this study.
Ethics oversight	All mouse procedures were carried out in strict accordance with the recommendations in the Guide for the Care and Use of Laboratory Animals of the National Institutes of Health. All animals were treated humanely. The protocol for the mouse chronic wound infection was approved by the Institutional Animal Care and Use Committee (IACUC) of Texas Tech University Health Sciences Center (Protocol Number 07044) and by the IACUC of Georgia Institute of Technology (Protocol Number A100127). The protocol for the mouse subcutaneous infection model was approved by the by the IACUC of Georgia Institute of Technology (Protocol Number A100127). For the mouse pneumonia infection model, all experiment procedures were conducted according to the guidelines of the Emory University Institutional Animal Care and Use Committee (IACUC), under approved protocol number DAR-201700441.

Note that full information on the approval of the study protocol must also be provided in the manuscript.
